# Identification of histone deacetylase inhibitors as neutrophil recruitment modulators in zebrafish using a chemical library screen

**DOI:** 10.1242/dmm.050056

**Published:** 2023-10-13

**Authors:** Sijia Fan, Jinlong Jiang, Huan Zhang, Cuihong Wang, Shang Kong, Tingting Zhao, Ling Meng, Yang Liu, Jingjing Qin, Xiuqin Rong, Zhenting He, Qinke He, Ke He, Ketong Chen, Ling Lei, Xinyu Hai, Hong Nie, Chunguang Ren

**Affiliations:** ^1^ Laboratory of Developmental Biology, Department of Cell Biology and Genetics, School of Basic Medical Sciences, Chongqing Medical University, Chongqing 400016, China; ^2^ International Cooperative Laboratory of Traditional Chinese Medicine Modernization and Innovative Drug Development of Chinese Ministry of Education (MOE) and Guangdong Province Key Laboratory of Pharmacodynamic Constituents of TCM and New Drugs Research, College of Pharmacy, Jinan University, Guangzhou 510632, China

**Keywords:** Zebrafish, Neutrophil, Cell migration, HDAC, AR-42

## Abstract

Tissue injury-induced neutrophil recruitment is a prerequisite for the initiation and amplification of inflammatory responses. Although multiple proteases and enzymes involved in post-translational modification (PTM) of proteins regulate leukocyte recruitment, an unbiased functional screen of enzymes regulating inflammatory leukocyte recruitment has yet to be undertaken. Here, using a zebrafish tail fin amputation (TFA) model to screen a chemical library consisting of 295 compounds that target proteases and PTM enzymes, we identified multiple histone deacetylase (HDAC) inhibitors that modulate inflammatory neutrophil recruitment. AR-42, a pan-HDAC inhibitor, was shown to inhibit neutrophil recruitment in three different zebrafish sterile tissue injury models: a TFA model, a copper-induced neuromast damage and mechanical otic vesicle injury (MOVI) model, and a sterile murine peritonitis model. RNA sequencing analysis of AR-42-treated fish embryos revealed downregulation of neutrophil-associated cytokines/chemokines, and exogenous supplementation with recombinant human IL-1β and CXCL8 partially restored the defective neutrophil recruitment in AR-42-treated MOVI model fish embryos. We thus demonstrate that AR-42 non-cell-autonomously modulates neutrophil recruitment by suppressing transcriptional expression of cytokines/chemokines, thereby identifying AR-42 as a promising anti-inflammatory drug for treating sterile tissue injury-associated diseases.

## Teaser

**Summary:** Multiple histone deacetylase inhibitors were identified from a chemical library screen to suppress neutrophil recruitment in a zebrafish injury model. AR-42 modulates neutrophil recruitment by suppressing the transcriptional expression of *il1b/cxcl8*.

## INTRODUCTION

It is well established that the inflammatory response functions as a defense mechanism that has evolved in higher organisms to protect them from infection and injury, and has recently emerged as an important regulator in homeostatic processes; however, excessive inflammation can result in pathological outcomes (Antonelli and Kushner, 2017; Gusev and Zhuravleva, 2022; Medzhitov, 2021). Inflammatory responses typically involve four consecutive steps, namely, inflammatory inducer presentation, sensor detection, inflammatory signals production by the sensor, and effector cell functioning (Medzhitov, 2008). Inflammation can be induced by tissue damage resulting either from sterile tissue injury or infection (the retrospective mode of induction), or by bacterial or viral components/products prior to tissue damage (the prospective mode of induction). These contrasting types of inflammatory induction are characterized by both common and differential mechanisms of inflammation recognition and subsequent immune modulation mediated by cytokines and effector cells (Medzhitov, 2021). During sterile tissue injury, damaged and dying cells produce or release damage-associated molecular patterns (DAMPs), such as high mobility group box 1 (HMGB1) protein, ATP, S100 proteins, mitochondrial formylated peptides and mitochondrial DNA. These DAMPs are sensed by a range of innate immune receptors, referred to as pattern recognition receptors, located on immune sentinel cells, which include macrophages, monocytes, dendritic cells, endothelial cells and stromal cells. In turn, these sentinel cells release multiple inflammatory cytokines, including TNFα and IL-1, as well as reactive oxygen species (ROS), that contribute to initiating neutrophil recruitment that further amplifies the inflammatory response, which is important for tissue repair and regeneration (Gong et al., 2020; Pittman and Kubes, 2013; Zindel and Kubes, 2020). As a key effector cell type involved in initiating inflammatory responses, neutrophils infiltrate damaged tissues via the process of transvascular migration, also referred to as extravasation, followed by interstitial migration, a sequence of events that is conserved from zebrafish to humans (Powell et al., 2017; Shelef et al., 2013). This directional transvascular migration of neutrophils is dependent on the presence of an intravascular chemokine gradient, whereas a gradient of DAMPs released directly from the site of injury provides the most potent chemotactic cue for neutrophils that have entered the interstitium and are in close proximity to the target wound (McDonald and Kubes, 2011). Notably, as a DAMP, ATP has been found to be released from neutrophils to induce cell clustering, and neutrophils tend to migrate in a clustered formation throughout the interstitial space, a process also referred to as neutrophil swarming (Kienle and Lämmermann, 2016). Furthermore, using a zebrafish tail fin amputation (TFA) model, Niethammer and colleagues found that epithelial cell-produced hydrogen peroxide forms a tissue-scale gradient to mediate the interstitial migration of neutrophils (Niethammer et al., 2009). In addition, it has been demonstrated that tissue injury/necrosis-induced nuclear swelling functions as a mechanotransducer that senses damage and activates cytosolic phospholipase A2 (cPLA2) to mediate the synthesis of pro-inflammatory eicosanoids, which serve as chemoattractants for neutrophil recruitment in a zebrafish TFA model (Enyedi et al., 2016; Katikaneni et al., 2020). However, although an extensive range of studies conducted to date have largely elucidated the molecular mechanisms underlying neutrophil recruitment into injured tissue (Pittman and Kubes, 2013), novel regulators in this process remain to be identified.

The zebrafish has emerged as a major model organism for the study of human diseases, including cancers and hematopoietic, cardiovascular, metabolic, immune and organ-specific diseases (Choi et al., 2021; Dooley and Zon, 2000; Lieschke and Currie, 2007). As vertebrates, zebrafish show high conservation with humans in terms of genetics and organ development. In contrast to mammals, the transparent zebrafish embryos develop externally, and numerous zebrafish leukocyte-specific transgenic reporter lines have been established and widely used (Choi et al., 2021). The *in vitro* fertilization and development, small size (1-2 mm for embryos and 3-4 cm for adult fish) and high fecundity (200-400 offspring/week/adult parents) of zebrafish are conducive to high-throughput *in vivo* drug screening using these fish (Lam and Peterson, 2019; Patton et al., 2021; Zon and Peterson, 2005). Importantly, it has been established that there is a clear temporal separation between the innate and adaptive immune responses in zebrafish, whereby only the innate immune system is functional during the first month of their lifespan (Novoa and Figueras, 2012). This unique advantage identifies zebrafish as a suitable system for studying the vertebrate innate immune response *in vivo*, independently of the adaptive immune response. Accordingly, zebrafish-based studies make a significant contribution to inflammatory disease modeling and anti-inflammatory drug development (Xie et al., 2020; Zanandrea et al., 2020). The zebrafish embryonic TFA model is a well-established acute tissue injury-induced sterile inflammatory model, which is widely used in the study of leukocyte recruitment and drug screening (Xie et al., 2020), and several studies have employed this model to screen for neutrophil recruitment suppressors in chemical libraries, including the Library of Pharmacologically Active Compounds (LOPAC) (Liu et al., 2013), the Prestwick Chemical Library (Hall et al., 2014) and other natural product libraries (Wang et al., 2014; Ye et al., 2015).

The migration of leukocytes is dependent on a network of molecules that include cytokines, chemokines and adhesion molecules. Notably, the synthesis, maturation and functioning of these molecules are all tightly controlled by proteolysis and post-translational modifications (PTMs) (Loh and Su, 2016; Smigiel and Parks, 2017; Van Lint and Libert, 2007; Vanheule et al., 2018). Proteases, such as matrix metalloproteinases, act beyond the extracellular matrix to modify cytokines, chemokines, antimicrobial peptides, surface proteins, receptors and junctional proteins, thereby regulating leukocyte activation and migration (Smigiel and Parks, 2017; Van Lint and Libert, 2007). However, although PTMs of cytoskeletal/adhesion-associated proteins, chemokines and their receptors play fundamental roles in the dynamic regulation of leukocyte migration (Loh and Su, 2016; Vanheule et al., 2018), an unbiased and appropriate scale of the functional screening of proteases and PTM enzymes that regulate inflammatory leukocyte recruitment remains to be reported. In this study, we performed a chemical screen for zebrafish neutrophil recruitment suppressors in a TFA model using a commercial chemical library comprising ∼300 chemicals that target proteases and PTM enzymes. Based on this screen, we identified multiple histone deacetylase (HDAC) inhibitors (HDACIs) as a representative cluster of hit compounds. By performing transcriptomic analysis, we further found that treatment with AR-42 (*N*-hydroxy-4-{[(2S)-3-methyl-2-phenylbutanoyl] amino} benzamide) downregulates the expression of cytokines/chemokines, and thereby contributes to the suppression of neutrophil recruitment.

## RESULTS

### HDACIs suppress tissue injury-induced neutrophil recruitment

**Fig. 1.**
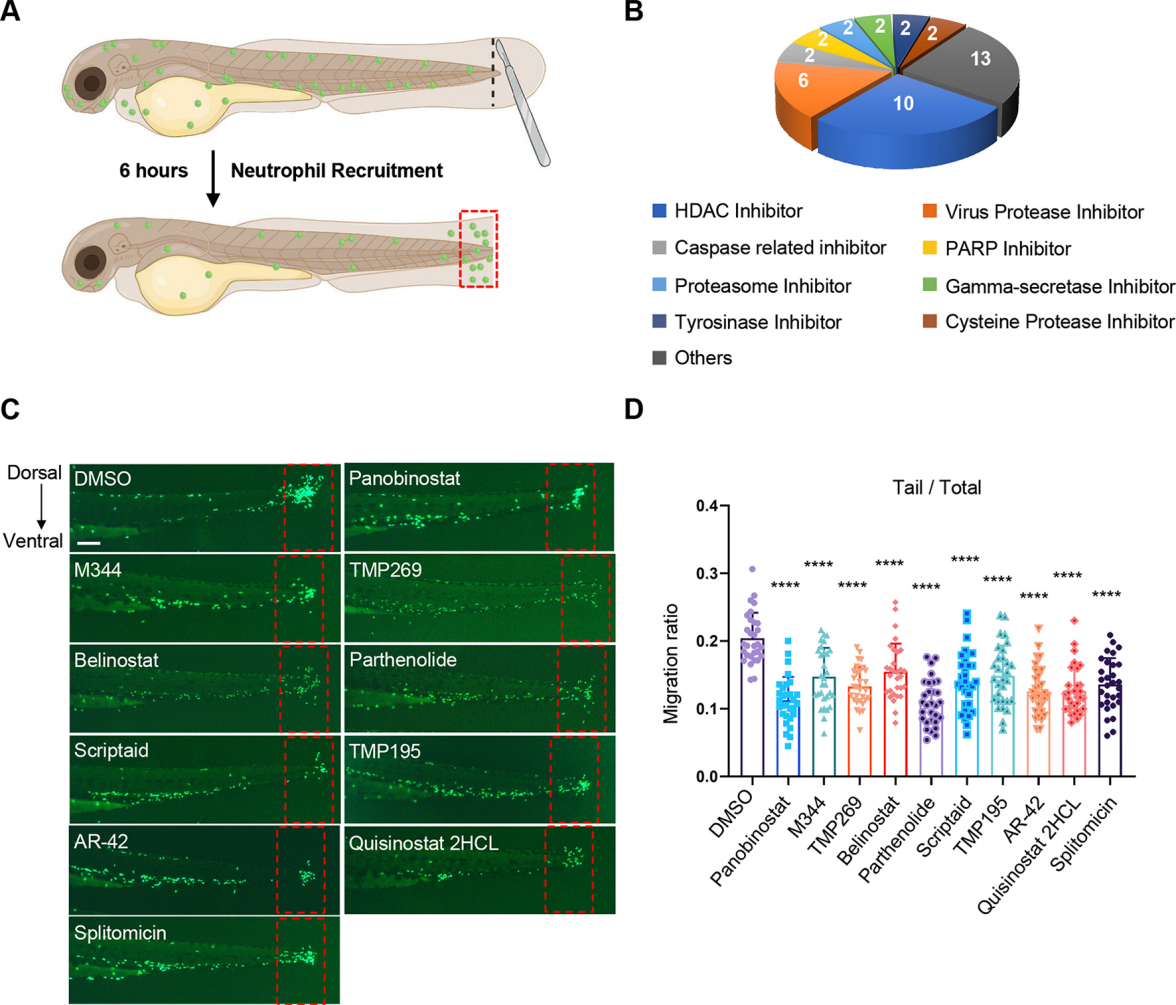
**HDACIs suppress tissue injury-induced neutrophil recruitment.** (A) Schematic diagram of the 3-day-post-fertilization (dpf) *Tg(mpx:GFP)^i114^* zebrafish embryonic tail fin amputation (TFA) model with imaging at 6 h post amputation. The diagram was created with BioRender.com. (B) Classification of 41 of 295 inhibitors targeting proteases and post-translational modification (PTM) enzymes that significantly inhibited neutrophil recruitment in zebrafish TFA model. (C) Representative fluorescence images of 3-dpf *Tg(mpx:GFP)^i114^* embryos treated with ten HDACs inhibitors from the library that suppressed zebrafish neutrophil recruitment in the TFA model. The red dashed rectangles denote the areas used for neutrophil counting. Scale bar: 100 µm. (D) Quantitative analysis of neutrophil recruitment in C (*n*=30) (see Materials and Methods for details). Each data point represents an individual embryo. Each HDACI group was individually compared with the DMSO group. Error bars represent mean±s.d. ****P<0.0001 (one-way ANOVA with Dunnett's test). The experiments in C,D were repeated four times.

Neutrophil-specific transgenic zebrafish *Tg(mpx:GFP)^i114^* embryos [3 days post fertilization (dpf)] were used to create a TFA model (Fig. 1A), and fluorescence images of the zebrafish larvae were captured at 6 h post amputation. Using this TFA model, we analyzed the effects of 295 protease and PTM enzyme inhibitors (Fig. S1A) on tissue injury-induced neutrophil recruitment, and accordingly found 41 inhibitors that suppressed neutrophil recruitment to the wound sites (Fig. 1B; Table S1). Among these hit compounds, we identified ten HDACIs, namely, panobinostat, M344, TMP269, belinostat, parthenolide, scriptaid, TMP195, AR-42, quisinostat 2HCL and splitomicin, that significantly suppressed the proportion and number of neutrophils recruited to the tail (Fig. 1C,D; Fig. S1B). Notably, these HDACIs had no discernible effect on the total number of neutrophils present throughout the embryos (Fig. S1C), thereby indicating the absence of any pro-apoptotic effects of HDACIs in this TFA model.

HDAC enzymes are grouped into four classes, class I-IV (de Ruijter et al., 2003). To investigate whether HDAC inhibition acts in a class-specific manner to modulate neutrophil recruitment, we further examined the effects of the following class-specific HDACIs on neutrophil recruitment in the TFA model: class I HDACIs (entinostat and BG45), class IIa HDACIs (TMP269 and LMK235), class IIb HDACIs (CAY10603 and tubastatin A), class IV HDACIs (elevenostat and SIS17) and a class III sirtuin inhibitor (suramin). The results revealed that inhibitors from all four HDAC classes suppressed neutrophil recruitment in zebrafish (Fig. S1D,E), thus indicating the absence of any class-specific effects among the HDACIs.

Collectively, the aforementioned data indicated that HDACIs modulate the inflammatory recruitment of neutrophils in this zebrafish model of acute tissue injury-induced sterile inflammation. Notably, among the 41 hit compounds, we identified AR-42, a new pan-HDACI with a low IC_50_ value (30 nM) that is currently in clinical trials for the treatment of cancer (Bondarev et al., 2021). However, with the exception of two previous studies that have reported that AR-42 modulates pro-inflammatory cytokine expression (Liao et al., 2018; Qu et al., 2016), the immune regulatory role of this inhibitor remains largely unknown, and it has yet to be determined whether AR-42 regulates leukocyte recruitment. Accordingly, in this study, we sought to focus on AR-42, with the aim of gaining detailed insights into the functional role of HDACI in neutrophil recruitment and to elucidate the associated underlying mechanisms.

### The pan-HDACI AR-42 inhibits tissue injury-induced neutrophil migration in a dose-dependent manner in the zebrafish TFA model

**Fig. 2.**
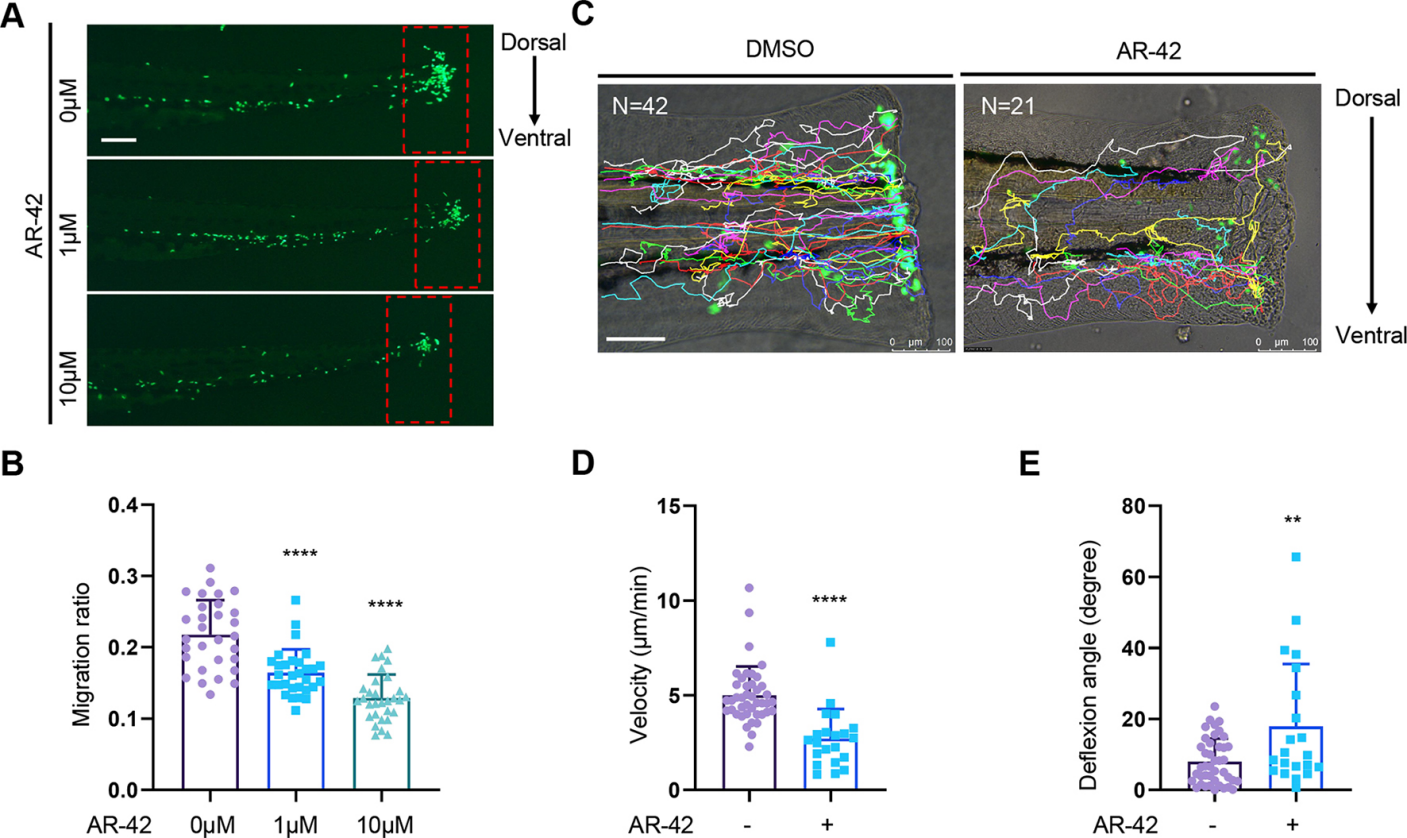
**Pan-HDACI AR-42 inhibits tissue injury-induced neutrophil migration in the zebrafish TFA model in a dose-dependent manner.** (A,B) Representative fluorescence images (A) and quantitative analysis (B) of neutrophil recruitment to the wound area of 3-dpf *Tg(mpx:GFP)^i114^* embryos treated with DMSO or AR-42 in the TFA model (*n*=30). Scale bar: 100 µm. The red dashed rectangles denote the areas used for neutrophil counting. Each data point represents an individual embryo (one-way ANOVA with Dunnett's test). The AR-42-treated group was individually compared with the DMSO group. (C) Representative migration trajectories of neutrophil chemotaxis toward the wound in DMSO- or 10 µM AR-42-treated 3-dpf *Tg(mpx:GFP)^i114^* embryos in the TFA model. Scale bar: 100 µm. ‘N’ denotes the number of GFP^+^ neutrophils that were tracked in one embryo. (D,E) Quantitative analysis of neutrophil migratory velocity (D) and deflexion angle (E) in C. Each data point represents an individual GFP^+^ neutrophil (two-tailed unpaired Student's *t*-test). Error bars represent mean±s.d. **P<0.01; ****P<0.0001. The experiments in A,B and C-E were repeated four and three times, respectively.

We found that AR-42 effectively inhibited neutrophil recruitment in the TFA model in a dose-dependent manner (Fig. 2A,B; Fig. S2A). Moreover, treatment with 10 μM AR-42 had no significant effect on either the number or distribution pattern of neutrophils in uninjured zebrafish embryos (Fig. S2B,C), thereby indicating that neutrophil differentiation and hemostasis are unaffected by AR-42. Furthermore, pre-treatment with 10 μM AR-42 for 4 h was observed to promote a marked reduction in the migration of neutrophils in zebrafish (Fig. S2D). To characterize this neutrophil migration behavior, we tracked the migrating neutrophils individually using TFA zebrafish embryos pre-treated with 10 μM AR-42 for 4 h. The results revealed that, compared with the control embryos, the AR-42-pre-treated embryos exhibited a slower velocity and reduced directionality (Fig. 2C-E), which was consistent with a non-significant slight reduction in the Euclidean distance (Fig. S2E). Additionally, we detected no significant difference in the accumulated distance (Fig. S2F). Collectively, these data indicate that AR-42 inhibits tissue injury-induced neutrophil migration in zebrafish in a dose-dependent manner.

We also examined the effects of AR-42 on macrophage recruitment. Interestingly, using a transgenic zebrafish macrophage reporter line, *Tg(mpeg1:Gal4)gl24;Tg(UAS:Nfsb-mCherry)i149*, we observed no appreciable influence of AR-42 treatment on macrophage hemostasis or recruitment to the wound (Fig. S3A-E), which indicates the specific inhibition of neutrophil migration by AR-42.

### AR-42 suppresses zebrafish neutrophil recruitment in copper-induced neuromast damage and mechanical otic vesicle injury models

**Fig. 3.**
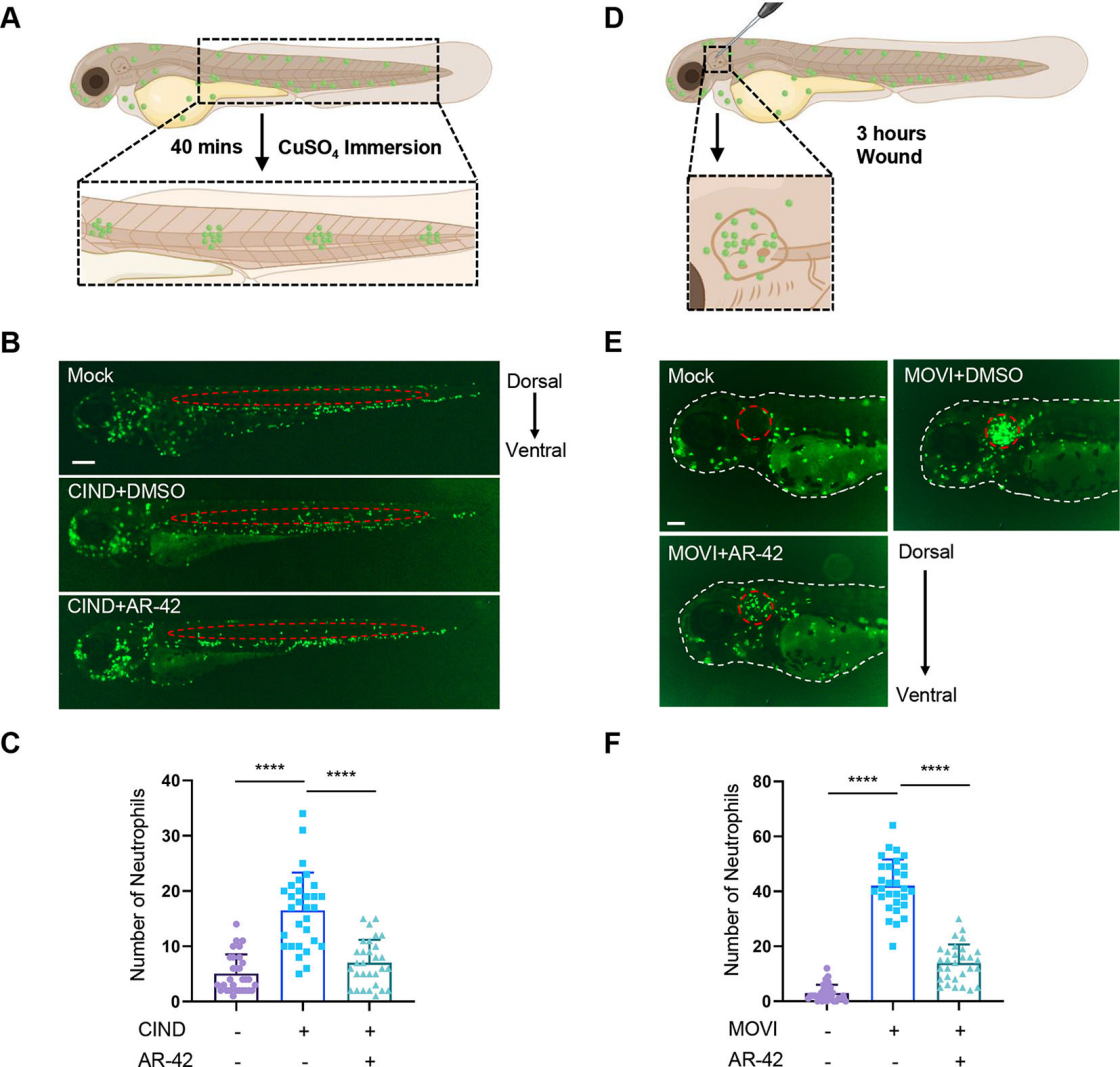
**AR-42 suppresses the zebrafish neutrophil recruitment in CIND and MOVI models.** (A) Schematic diagram of the copper-induced neuromast damage (CIND) model. (B) Representative fluorescence images of DMSO- or AR-42-treated 3-dpf *Tg(mpx:GFP)^i114^* embryos in the CIND model. Scale bar: 200 µm. The red dashed ovals denote the neuromast regions used for neutrophil counting. (C) Quantification of neutrophils recruited to zebrafish neuromasts in B (*n*=30). (D) Schematic diagram of the mechanical otic vesicle injury (MOVI) model. (E) Representative fluorescence images of DMSO- or AR-42-treated 3-dpf *Tg(mpx:GFP)^i114^* embryos in the MOVI model. Scale bar: 100 µm. The white dashed lines denote the contours of the embryos. The red dashed circles denote the otic vesicle contours. (F) Quantification of neutrophils recruited to the zebrafish otic vesicle in E (*n*=30). Each data point represents an individual embryo (one-way ANOVA with Dunnett's test) in C,F. Error bars represent mean±s.d. ****P<0.0001. The experiments in B,C,E,F were repeated four times.

We further examined whether AR-42 exhibited a broad inhibitory effect on neutrophil recruitment using two different aseptic zebrafish tissue injury models. d'Alençon et al. (2010) have demonstrated that exposure of zebrafish larvae to sublethal concentrations of copper sulfate selectively damages the sensory hair cell population and induces infiltration of leukocytes to neuromasts within 20 to 40 min, and we accordingly established a chemical-induced zebrafish tissue injury model, referred to as the copper-induced neuromast damage (CIND) model, which can be used to quantitatively analyze neutrophil recruitment in zebrafish neuromasts at 3 dpf (Fig. 3A). The results revealed that, compared with DMSO treatment, AR-42 treatment effectively suppressed neutrophil recruitment to neuromast regions (Fig. 3B,C). Additionally, we established a novel quantitatively aseptic mechanical otic vesicle injury (MOVI) model to validate the role of AR-42 in modulating neutrophil recruitment, using which we demonstrated that a mechanical stab of the otic vesicle induced a rapid wave of neutrophil recruitment to the wounded otic vesicle within 1 h, with the neutrophil influx reaching a peak at approximately 3 h post injury (Fig. 3D; Fig. S4A,B). Consistent with observations using the CIND model, we found that AR-42 treatment also caused a reduction in neutrophil infiltration into the injured otic vesicle in the MOVI model (Fig. 3E,F). These results thus indicate that AR-42 broadly suppresses the inflammatory recruitment of neutrophils in multiple sterile tissue injury models.

### AR-42 inhibits neutrophil inflammatory recruitment in a sterile mouse peritonitis model

**Fig. 4.**
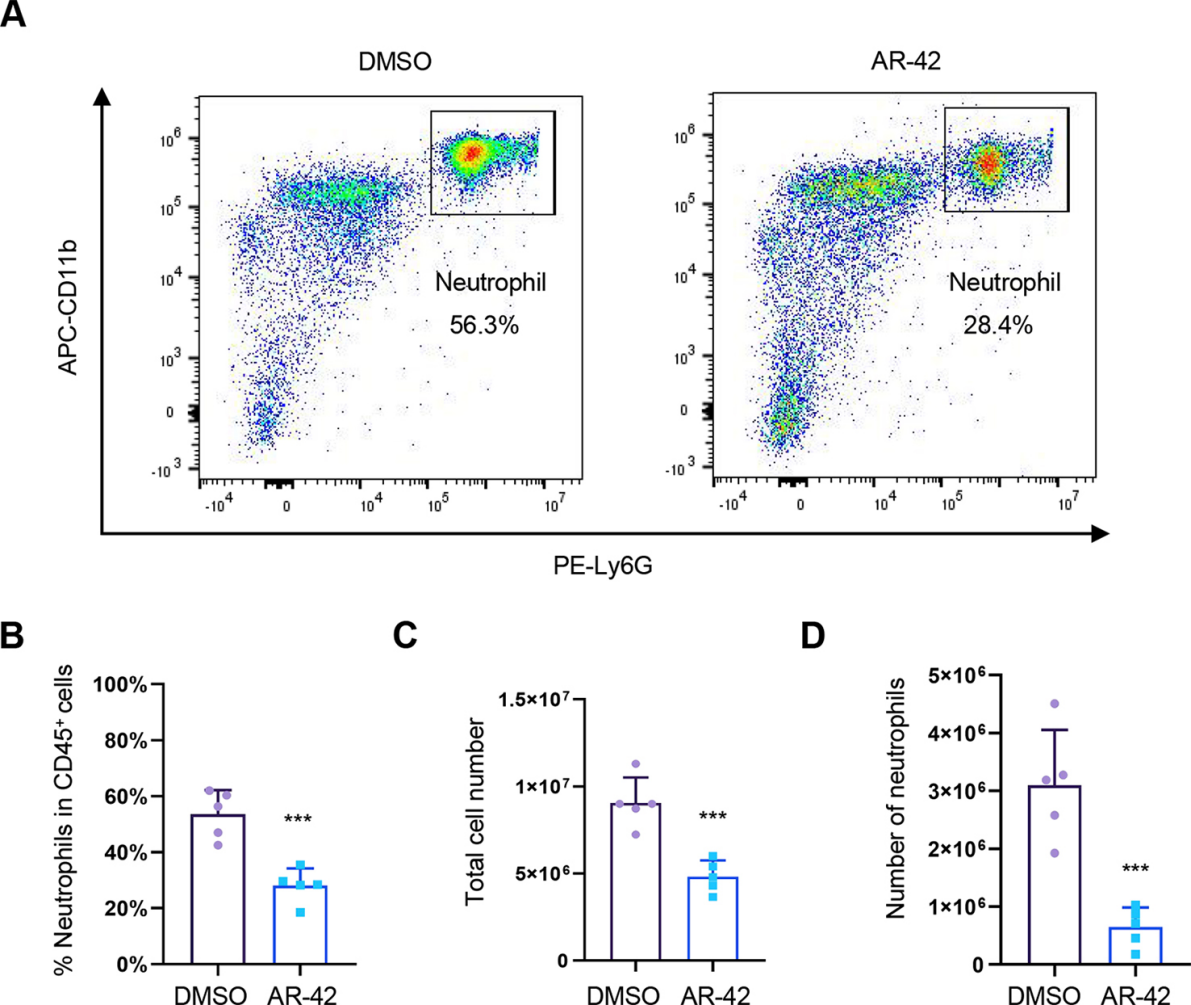
**AR-42 inhibits neutrophil inflammatory recruitment in the mouse peritonitis model.** (A) Representative dot plots of neutrophil percentages that were analyzed by flow cytometry. (B) Quantitative analysis of the percentage of neutrophils in CD45^+^ cells in peritoneal lavage fluid. (C) Quantitative analysis of total cell number in peritoneal lavage fluid. (D) Quantitative analysis of the number of neutrophils in peritoneal lavage fluid. Each data point represents an individual mouse (two-tailed unpaired Student's *t*-test). Error bars represent mean±s.d. ***P<0.001. The experiments in A-D were repeated three times.

Having demonstrated that AR-42 suppresses neutrophil recruitment in sterile zebrafish tissue injury models, we further investigated whether AR-42 also affects mouse neutrophil recruitment *in vivo* by using an autoclaved thioglycollate medium-induced mouse peritonitis model, which is an acute inflammatory model characterized by predominant neutrophil infiltration. The results revealed that the injection of AR-42 promoted a robust attenuation of neutrophil infiltration into the inflamed peritoneal cavity (Fig. 4A-D), thereby providing evidence that AR-42 also influences neutrophil recruitment in a sterile mouse peritonitis model.

### AR-42 treatment downregulates the neutrophil migration-associated expression of cytokines/chemokines

Having functionally validated the role of AR-42 in *in vivo* neutrophil recruitment using multiple aseptic zebrafish and mouse tissue injury models, we subsequently sought to determine whether AR-42 acts on neutrophils to directly influence neutrophil migration (in a cell-autonomous manner) or whether this effect is mediated indirectly through other cells (in a non-cell-autonomous manner). To overcome the technical difficulties associated with studying the direct effects of AR-42 on neutrophil migration using *in vivo* tissue injury models, we alternatively investigated whether AR-42 directly modulates the migration of purified neutrophils *in vitro* using the Dunn chamber assay to assess the effects of AR-42 on the chemotaxis of mouse bone marrow-derived primary neutrophils. The results revealed that AR-42-pre-treated neutrophils were characterized by normal migratory velocity, directionality, accumulated distances and Euclidean distances (Fig. S5A-E), indicating that the inhibitory effects of AR-42 on neutrophil migration may be independent of its direct interaction with neutrophils. Consistently, we found that neutrophil spreading, a prerequisite for cell adhesion and migration, was unaffected by AR-42 treatment (Fig. S5F,G). Taken together, these results obtained using purified mouse primary neutrophils indicate that it is unlikely that AR-42 regulates neutrophil migration in a cell-autonomous manner.

**Fig. 5.**
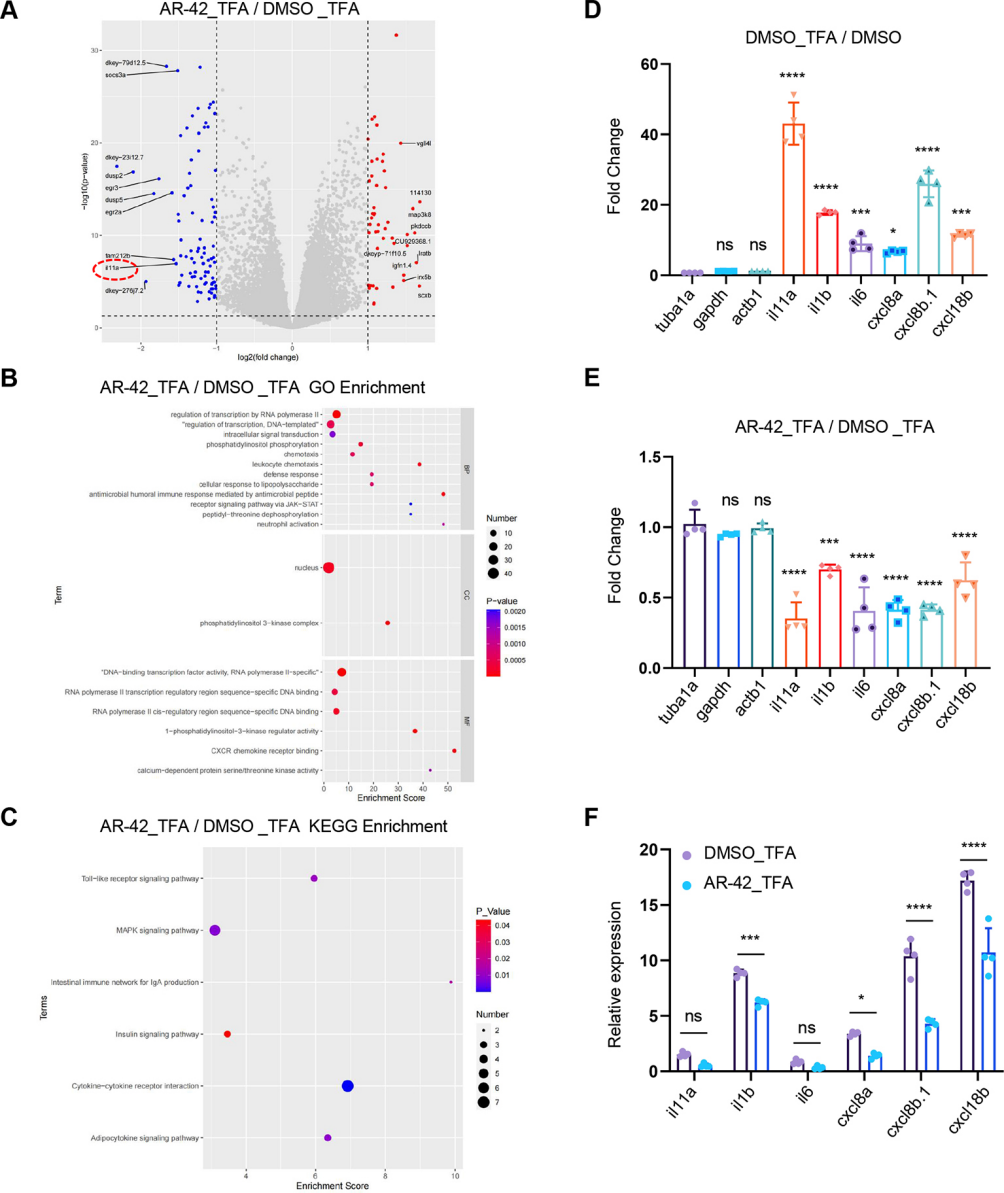
**AR-42 treatment downregulates neutrophil migration-associated cytokine/chemokine expression.** (A) Volcano plot of RNA-seq data with differentially expressed genes between the AR-42_TFA and DMSO_TFA groups (see Materials and Methods for details). The red dashed oval highlights *il11a,* which is among the top ten downregulated genes. (B) Differentially expressed related pathways between the AR-42_TFA and DMSO_TFA groups were analyzed by GO pathway enrichment. (C) Differentially expressed related pathways between the AR-42_TFA and DMSO_TFA groups were analyzed by KEGG pathway enrichment. (D,E) Fold change of neutrophil migration associated-cytokines/chemokines in the DMSO_TFA group compared to the DMSO group (D) and in the AR-42_TFA group compared to the DMSO_TFA group (E). The fold change was normalized to *tuba1a* expression. Each data point represents a biological replicate (one-way ANOVA with Dunnett's test). (F) FPKM values of neutrophil migration associated-cytokines/chemokines in the DMSO_TFA and AR-42_TFA groups. Each data point represents a biological replicate (two-way ANOVA with Šídák's test). Data show the mean±s.d. ns, not statistically significant; *P<0.05; ***P<0.001; ****P<0.0001. The experiments in A-F were repeated four times.

**Fig. 6.**
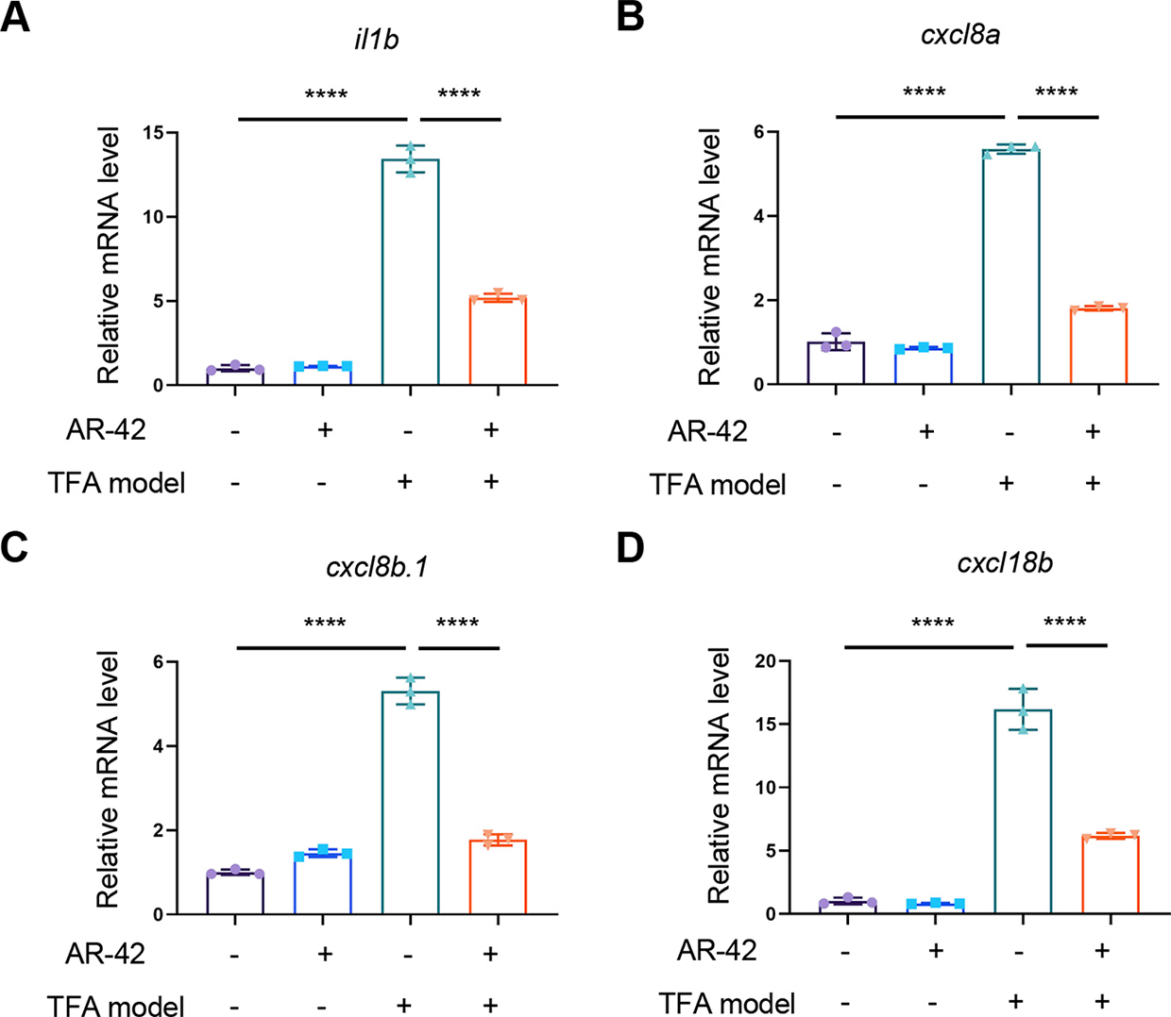
**AR-42 suppresses the expression of zebrafish *il1b*, *cxcl8a*, *cxcl8b.1* and *cxcl18b*.** (A-D) qRT-PCR analysis of the expression levels of *il1b* (A), *cxcl8a* (B), *cxcl8b.1* (C) and *cxcl18b* (D) in 3-dpf wild-type zebrafish embryos treated with DMSO or 10 µM AR-42 for 1 h with or without TFA modeling. Eighty larvae were used for each treatment. The relative expression was quantified by the 2-ΔΔct method. *tuba1a* was used as an internal control. Each data point represents a biological replicate (one-way ANOVA with Dunnett's test). Error bars represent mean±s.d. ****P<0.0001. The DMSO and AR-42_TFA groups were compared to the DMSO_TFA condition. The experiments in A-D were repeated three times.

We subsequently investigated the inflammatory environmental effects of AR-42 on neutrophil recruitment by performing RNA-sequencing (RNA-seq) analysis using tail tissue obtained from TFA model zebrafish. The RNA-seq results revealed that the cytokine *il11a* was among the top ten downregulated genes in the AR-42-treated tail tissue sample (Fig. 5A), implying that AR-42 modulates cytokine expression. Consistently, Gene Ontology (GO) enrichment analysis revealed a clear enrichment of ‘leukocyte chemotaxis’, ‘neutrophil activation’ and ‘CXCR chemokine receptor binding’ in the AR-42 group (Fig. 5B), and Kyoto Encyclopedia of Genes and Genomes (KEGG) enrichment analysis revealed a robust enrichment of ‘cytokine signaling’ in the AR-42 group (Fig. 5C). These observations thus prompted us to examine the expression of several neutrophil recruitment-associated cytokines/chemokines, including *il1b*, *il6*, *cxcl8a*, *cxcl8b.1* and *cxcl18b*, in our RNA-seq data. The results revealed that the expression of all these genes was stimulated in response to TFA (Fig. 5D), but significantly attenuated by AR-42 treatment (Fig. 5E). Additionally, we found that *il1b*, *cxcl8a*, *cxcl8b.1* and *cxcl18b* exhibited relatively high expression (Fig. 5F), and these genes were accordingly selected for quantitative real-time PCR (qRT-PCR) validation. The qRT-PCR results revealed that the mRNA levels of *il1b*, *cxcl8a*, *cxcl8b.1* and *cxcl18b* were substantially induced by TFA modeling and significantly reduced upon AR-42 treatment (Fig. 6A-D), which was consistent with our RNA-seq data. These findings thus indicate that AR-42 modulates cytokine/chemokine signaling in the local inflammatory environment rather than directly modulating neutrophil migration.

### Exogenous supplementation of recombinant human proteins IL-1β and CXCL8 partially rescues the defective neutrophil recruitment upon AR-42 treatment

**Fig. 7.**
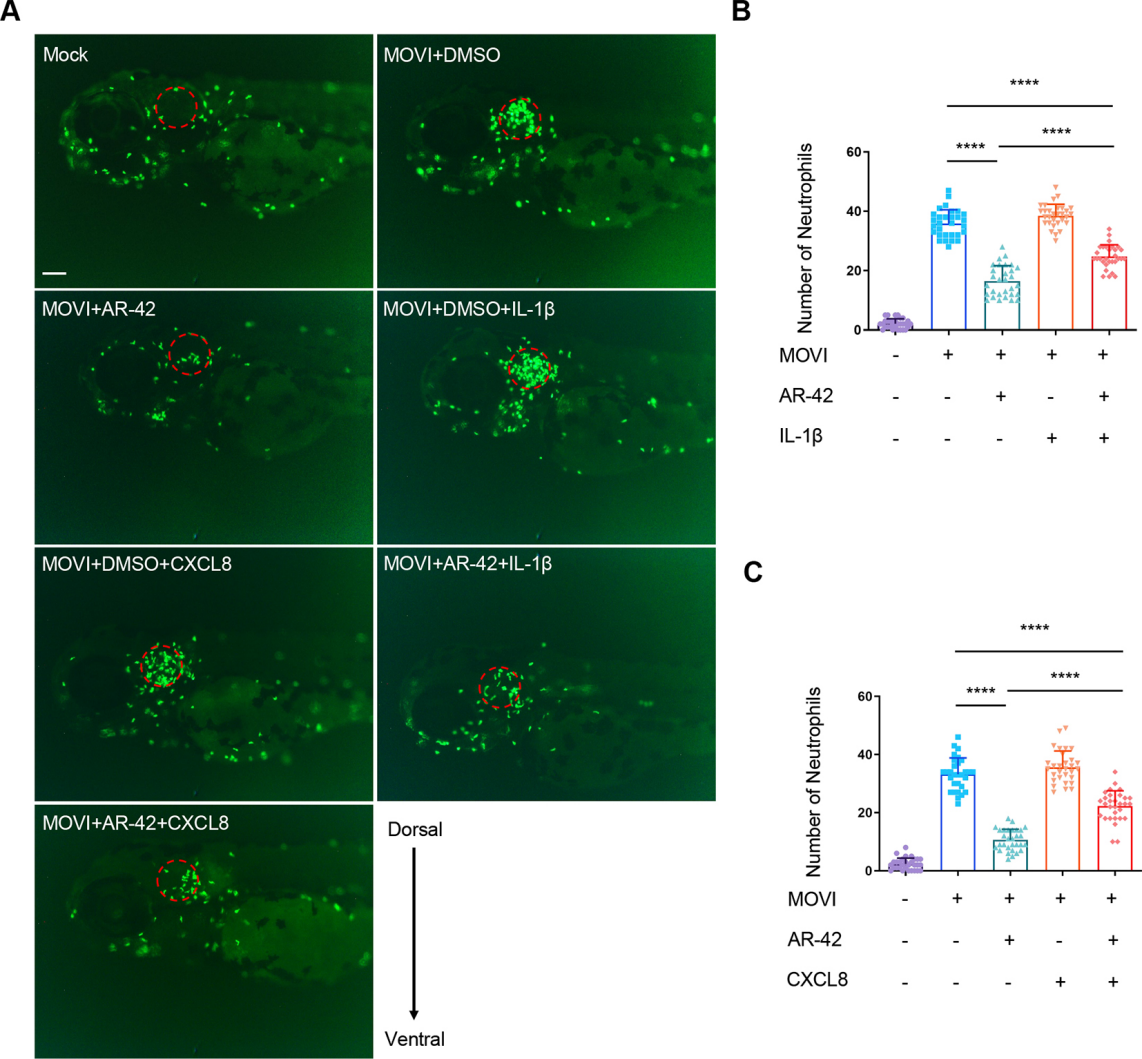
**Exogenous supplementation of recombinant human IL-1β and CXCL8 partially rescues the defective neutrophil recruitment upon AR-42 treatment.** (A-C) Representative fluorescence images (A) and quantitative analysis (B,C) of neutrophil recruitment to the otic vesicle in DMSO- or AR-42-treated 3-dpf *Tg(mpx:GFP)^i114^* embryos with or without recombinant human IL-1β/CXCL8 proteins injection in a MOVI model (*n*=30). Scale bar: 100 µm. The red dashed circles denote the otic vesicle contours. Each data point represents an individual embryo (one-way ANOVA with Tukey's test). Error bars represent mean±s.d. ****P<0.0001. The experiments in A-C were repeated three times.

Given that Il1b and Cxcl8 have previously been demonstrated to be essential for zebrafish neutrophil recruitment in the TFA model (de Oliveira et al., 2013; Yan et al., 2014), we further examined whether the downregulation of *il1b* and *cxcl8* induced by AR-42 is responsible for defective neutrophil recruitment by performing a functional rescue assay based on the exogenous supplementation of IL-1β and CXCL8 proteins. For this purpose, we adopted the zebrafish MOVI model rather than the TFA model based on the fact that the injection of exogenous protein into the hollow otic vesicle is more feasible than injection into the solid tail tissue. The results revealed that injection of either human recombinant IL-1β or CXCL8 protein was effective in rescuing the migration of neutrophils reduced by AR-42 treatment (Fig. 7A-C), indicating functional conservation between human and zebrafish cytokines/chemokines, even though the protein sequences show relatively weak homology. This functional conservation between human and zebrafish IL-1 signaling was further confirmed by our observations that injection of the recombinant human IL-1β into zebrafish otic vesicles stimulated the expression of IL-1 target genes, such as *nfkbiaa*, *nfkbiab*, *ccl2*, *cxcl8a*, *cxcl8b.1*, *ptgs2a*, *ptgs2b* and *il1b*, in fish (Fig. S6A-H) (Weber et al., 2010). Notably, *nfkbiaa*, *nfkbiab*, *ccl2*, *cxcl8a*, *cxcl8b.1* and *il1b*, but not *ptgs2a* or *ptgs2b*, were not upregulated by the injection of control proteins including myoglobin or ENSA (Fig. S6A-H), which is generally unrelated to IL-1β signaling, thus largely precluding a non-specific response to proteins. Collectively, these results indicate that AR-42 suppresses neutrophil recruitment *in vivo* by modulating the transcriptional expression of neutrophil migration-associated cytokines/chemokines, including *il1b* and *cxcl8*.

## DISCUSSION

In this study, we used a chemical library comprising 295 compounds with known targets to screen for neutrophil recruitment modulators using a sterile zebrafish TFA model. Among the numerous HDACIs identified as hits compounds, a pan-HDACI, AR-42, was shown to downregulate neutrophil migration-associated cytokine/chemokine expression. In addition, we demonstrated that exogenous supplementation of IL-1β and CXCL8 can rescue defective neutrophil recruitment in AR-42-treated zebrafish embryos. Notably, we also identified several hit chemicals that have previously been reported to suppress neutrophil migration, including tanshinone IIA (Liu et al., 2016), ibuprofen (Bertolotto et al., 2014) and calycosin (Cheng et al., 2015), which provides further evidence highlighting the efficacy of our screening protocol.

HDACs comprise a large family of epigenetic metalloenzymes responsible for regulating the expression of an extensive array of genes by mediating the reversible deacetylation of nuclear histones, as well as a large number of non-histone proteins involved in gene transcription regulation, thus playing important roles in cell proliferation, differentiation, migration and death (de Ruijter et al., 2003). HDACs have also been established to be involved in neutrophil migration and recruitment. For example, HDAC2 has been shown to negatively regulate the trans-endothelial migration of neutrophil-like HL60 cells and neutrophil recruitment in a murine model of acute ischemic stroke (Li et al., 2020), whereas HDAC11-deficient neutrophils have been demonstrated to have an enhanced chemotactic capacity (Sahakian et al., 2017). In the present study, we showed that both pan-HDACIs and subfamily-specific HDACIs sufficiently inhibited tissue injury-induced neutrophil recruitment in zebrafish *in vivo*. Additionally, we found that AR-42-treated mouse primary neutrophils exhibited normal chemotaxis. These findings thus indicate an inflammatory environmental effect on neutrophil recruitment by AR-42, which is distinct from the cell-autonomous effects of HDAC2/11 on neutrophil migration. Notably, our observations are consistent with the findings of a previous study that reported a reduction in inflammatory neutrophil recruitment, a reduction in the expression of cytokines, including TNFα and IL-1β, and the amelioration of pathogenic phenotypes in HDACI-treated murine inflammatory disease models, including lipopolysaccharide (LPS)-induced acute lung injury (Ni et al., 2010), LPS-induced endometritis (Guo et al., 2019), cigarette smoke-induced airway inflammation (Leus et al., 2017), spinal cord injury (Zhang et al., 2018) and aristolochic acid-induced kidney injury (Novitskaya et al., 2014). Taken together, these findings indicate that HDACIs play protective roles in both septic and aseptic tissue injury models, although it remains to be determined whether the mechanisms of action whereby HDACIs mediate these effects in the two injury model types are the same.

To date, several HDACIs have been clinically approved for the treatment of both leukemia and solid cancers, based on the suppression of the viability and proliferation of cancer cells (Ho et al., 2020). Interestingly, HDACIs are also emerging as effective therapeutic agents for a spectrum of diseases unrelated to cancer, among which are neurodegenerative disorders, HIV-1 purging, and autoimmune and chronic inflammatory diseases (Dinarello et al., 2011). Two HDACIs, vorinostat (SAHA) and ITF2357, are currently undergoing clinical trials for the treatment of graft-versus-host disease (GVHD) (Perez et al., 2021) and active systemic juvenile idiopathic arthritis (SOJIA) (NCT00570661), respectively. Distinct from their use in cancer, a reduction in inflammation promoted by HDACIs is consistently observed at low concentrations compared with the higher concentrations required for the killing of tumor cells (Hull et al., 2016). This characteristic makes HDACIs attractive candidates for the treatment of inflammatory diseases, as low doses tend to be well tolerated. Compared with SAHA, AR-42 has been shown to have significantly superior apoptotic efficacy (Murahari et al., 2017) and, indeed, is currently being assessed in clinical trials for the treatment of relapsed multiple myeloma (NCT02569320), acute myeloid leukemia (NCT01798901), chronic lymphocytic leukemia (NCT01129193) and meningiomas (NCT05130866). Notably, however, AR-42 has not yet been investigated with respect to its role in immune regulation. In this regard, the administration of AR-42 has been reported to suppress the transcriptional expression of pro-inflammatory cytokines, such as IL-1β, TNFα and IL-6, in trinitrobenzene sulfonic acid-induced chronic pancreatitis in mice (Liao et al., 2018). A further study has reported that *in vitro* treatment using AR-42 enhances the secretion of IL-1β, TNFα and IL-6 in monocytes obtained from patients with gestational diabetes mellitus (Qu et al., 2016). In the present study, using zebrafish injury models, we demonstrated that AR-42 suppresses the mRNA expression of these cytokines for immune modulation in sterile tissue injury. The immune inhibitory role of AR-42 identified in this study is consistent with previous findings reported by Liao et al. (2018). Notably, GVHD and SOJIA, which are being clinically targeted using HDACIs as mentioned above, are generally considered as aseptic tissue/organ damage-associated diseases with the involvement of alloimmune or autoimmune mechanisms, thereby highlighting the clinical therapeutic potential of HDACIs in sterile tissue injury. In this study, we identified that AR-42 suppresses neutrophil recruitment in sterile zebrafish tissue injury models, and thus, in addition to vorinostat (SAHA) and ITF2357, our findings may provide a basis for the development of AR-42 as an effective novel HDACI for the treatment of tissue injury-associated diseases.

Given that AR-42 was found to non-cell-autonomously regulate neutrophil recruitment, we sought to investigate whether AR-42 modulates neutrophil migration indirectly via the modulation of cytokine/chemokine signaling. The results of our transcriptomic analysis of injured fish tail tissues indeed revealed enrichment of cytokine signaling transcripts. Most of the neutrophil migration-associated cytokines and chemokines detected in our RNA-seq data were significantly downregulated upon AR-42 treatment, a pattern that was subsequently verified by qRT-PCR analysis. AR-42 treatment was observed to suppress the expression of *il1b*, *cxcl8* and *cxcl18*, whereas exogenous injection of recombinant human IL-1β and CXCL8 was effective in, at least partially, rescuing the defective neutrophil recruitment attributable to AR-42. Consistent with the findings of previous studies (de Oliveira et al., 2013; Yan et al., 2014), from a functional perspective, our findings confirm the important roles played by Il1b and Cxcl8 in neutrophil recruitment. In zebrafish, it has been established that Cxcl8 acts downstream of the Il1b-Myd88 axis to regulate injury-induced neutrophil migration (Yan et al., 2014). However, the upstream regulatory mechanism for the stimulation of *il1b* expression upon sterile injury remains unknown, and it is at least not regulated by NADPH oxidase-mediated ROS production (Yan et al., 2014). Notably, IL-1β (Yan et al., 2014) and NADPH oxidase-mediated ROS (Niethammer et al., 2009; Yoo et al., 2011) have been implicated to independently and differentially regulate neutrophil recruitment in the zebrafish TFA model (Yan et al., 2014). Whether AR-42 modulates ROS production needs further investigation, nevertheless, our study strongly suggests that HDACs act upstream of the Il1b-Myd88 axis to dictate neutrophil recruitment.

Although we did not determine the mechanisms whereby AR-42 modulates *il1b* expression in this study, it has been established that HDACs regulate the expression of multiple cytokines and chemokines via PTM activity (Gatla et al., 2019). HDACIs regulate the immune response by influencing the acetylation of histone and non-histone proteins (e.g. p53, GATA1-3, STAT3, STAT5, Foxp3 and NF-κB) (Hull et al., 2016; Kim et al., 2016), indicating the involvement of both chromatin remodeling-dependent and -independent mechanisms in the regulation of these proteins. HDACIs have been shown to attenuate the LPS-stimulated expression of *IL1B* in epithelial (KB31), fibroblast (3T3-J2) and myogenic (C2C12) cells (Di Liddo et al., 2016), and the HDACI trichostatin A has been found to suppress TNFα expression in LPS-stimulated cardiomyocytes by enhancing acetylation of the p65 subunit of NF-κB (Zhu et al., 2010), which has been suggested to attenuate NF-κB transcriptional activity (Kiernan et al., 2003). Additionally, a reduction in pro-inflammatory cytokine levels and NF-κB signaling has previously been observed in HDACI-treated inflammatory disease models (Guo et al., 2019; Ni et al., 2010). Thus, given these previous findings, it would be of interest to determine whether the inhibition of HDACs by AR-42 promotes the downregulated expression of cytokines such as *Tnfa* and *Il1b* by inducing chromatin remodeling or modulating the acetylation of the p65 subunit of NF-κB. Although chromatin remodeling has been established to be essential for neutrophil activation and inflammatory gene transcriptional profiling (Denholtz et al., 2020), our mouse neutrophil data indicate that AR-42 is unlikely to act directly on neutrophils to regulate their recruitment. Nevertheless, it would be necessary to examine whether AR-42 treatment has a direct influence on neutrophil activation via its chromatin remodeling activity. A further important issue concerns identifying the types of cells directly targeted by AR-42, as well as the cellular source of IL-1β production in our model, for which further studies are certainly warranted.

In summary, based on a chemical library screen, we demonstrated that the pan-HDACI AR-42 suppresses sterile tissue injury-induced neutrophil recruitment in zebrafish by modulating the expression of *il1b* and *cxcl8*. Although more detailed mechanistic studies are necessary to further consolidate these findings, this study is, to the best of our knowledge, the first to elucidate the function of AR-42 in regulating leukocyte migration, thereby identifying AR-42 as a promising anti-inflammatory agent for the treatment of sterile tissue injury-associated diseases.

## MATERIALS AND METHODS

### Zebrafish and mice

Transgenic zebrafish lines [*Tg(mpx:GFP)^i114^* (Renshaw et al., 2006) and *Tg(mpeg1:Gal4)gl24;Tg(UAS:Nfsb-mCherry)i149* (Ellett et al., 2011)] were raised and kept under standard conditions. All fish were maintained in a circulation culture system of 28±1°C, with a photoperiod of 14 h:10 h light:dark, and fed with Artemia. All embryos were collected through natural spawning and raised at 28.5°C in Petri dishes containing E3 medium (5 mM NaCl, 0.17 mM KCl, 0.33 mM CaCl_2_, 0.33 mM MgSO_4_, with 0.01% Methylene Blue and equilibrated to pH 7.0).

The C57BL/6N wild-type mice were purchased from Charles River Laboratories. Mice were kept in group housing at a temperature of 25°C with a photoperiod of 12 h:12 h light:dark and were given unlimited access to potables and standard mouse food. Eight- to 12-week-old females were used for bone marrow-derived primary neutrophil isolation.

All animal studies were approved by the Institutional Animal Care and Use Committees of Chongqing Medical University.

### Chemicals and reagents

The TargetMol Protease Inhibitor Library consisting of 295 compounds and the inhibitors of HDAC classes I-IV were purchased from Topscience (China, L1100). All inhibitor powders were dissolved in DMSO to a concentration of 10 mM in stock solutions and stored at −20°C, and the working concentration of chemicals for the screen was 10 µM.

### Zebrafish larvae TFA model

As previously described (Bernut et al., 2020; Zanandrea et al., 2020), 3-dpf *Tg(mpx:GFP)^i114^* or *Tg(mpeg1:Gal4)gl24;Tg(UAS:Nfsb-mCherry)i149* larvae were anesthetized with tricaine and arranged in agarose-coated dishes for amputation of the tail fin. The posterior portion of the ventral pigmental gap of the caudal fin was used as an anatomical reference for transection under a stereomicroscope. Embryos 6 h post amputation were imaged under a ZEISS SteREO Discovery (V12) fluorescence microscope. Neutrophil chemotaxis was assessed by counting GFP^+^ cells in the whole embryo (referred to as ‘total’) as well as GFP^+^ cells recruited to the wound area (referred to as ‘tail’). Migration efficiency was defined as a ‘tail’/’total’ ratio in our study.

For the chemical library screening, 3-dpf *mpx:GFP* larvae were subjected to TFA modeling and then ten larvae were immediately arrayed into each well of the 12-well plate. In each well, 3 ml E3 medium containing one compound from the library at the concentration of 10 µM was added. A negative control of DMSO (3 µl per well)-treated larvae was included in each plate. After a 6 h incubation at 28.5°C, larvae were imaged and quantified as mentioned above. The potential hit compounds obtained from the initial screening were subjected to three independent replicative experiments with increasing sample numbers (*n*=30) to confirm their suppressive effects on neutrophil recruitment in zebrafish TFA model.

### Time-lapse analysis of zebrafish neutrophils migration *in vivo*

The 3-dpf *Tg(mpx:GFP)^i114^* transgenic zebrafish larvae were pretreated with DMSO or 10 μM AR-42 for 4 h followed by TFA modeling. Then, the injured larvae were anesthetized with tricaine and mounted in 1% low-melting agarose in a Petri dish for time-lapse imaging under a Leica DMI8 microscope. All time-lapse image series were acquired at 1 min intervals for 6 h and analyzed using the ImageJ plugin ‘Chemotaxis And Migration Tool’ (Schindelin et al., 2012). We chose neutrophils appearing within 100-550 µm anterior to the tail end to start tracking to preclude randomly migrating neutrophils.

We measured four chemotactic parameters, namely, directionality velocity, deflexion angle, Euclidean distance and accumulated distance, as mentioned previously (Ren et al., 2019). The deflexion angle represents the angle between the direction of the straight line and the direction of cell migration. The accumulated distance is the route of neutrophil migration, whereas the Euclidean distance, also known as displacement, is the distance traveled in a straight line by moving cells. The straight-line migration distance from the origin divided by the total migration length is how directionality was calculated. The average cell migratory speed (accumulated distance/total time) was used to compute velocity (μm/min).

### CIND model

The CIND model was established as previously reported (d'Alençon et al., 2010). Briefly, 3-dpf *Tg(mpx:GFP)^i114^* transgenic larvae were treated with 10 μM CuSO_4_ for 40 mins at 28°C. The larvae were then fixed with 4% paraformaldehyde for 1 h at room temperature, followed by imaging under a ZEISS SteREO Discovery (V12) fluorescence stereoscope. We counted GFP^+^ cells within ten cell diameters of the horizontal myoseptum between the first somite and the end of the tail in each larva, and this region included the L1-L5 neuromasts in the posterior lateral line while excluding the two to three terminal neuromasts.

### Zebrafish MOVI model and recombinant protein supplementation assay

In this study, we created a novel quantitatively aseptic MOVI model to provide an alternative mechanical injury model for studying fish neutrophil recruitment *in vivo*. The 3-dpf *Tg(mpx:GFP)^i114^* zebrafish larvae were anesthetized and the otic vesicle was repeatedly stabbed five times with a hollow microinjection needle (borosilicate glass, BF100-78-10) with a diameter of ∼50 μm at the tip using a Warner microinjection system (PLI-90A). Immediately after trauma, the larvae were incubated with DMSO or 10 µM AR-42 for the indicated time before imaging under a ZEISS SteREO Discovery (V12) fluorescence stereomicroscope. Then the neutrophils recruited to the otic vesicle site were counted for quantitative analysis.

For the rescue experiment, we injected 1 nl human recombinant protein IL-1β (0.1 mg/ml, Peprotech, 200-01B) or CXCL8 (0.1 mg/ml, Peprotech, 200-08) into the injured otic vesicle immediately after MOVI modeling, and 3 h post injection, microscopic imaging and counting were performed. For the expression analysis of zebrafish IL-1 target genes by the human IL-1β protein, we injected 1 nl sterile H_2_O or recombinant human IL-1β (0.1 mg/ml), myoglobin (0.1 mg/ml, MedChemExpress, HY-P70243) or ENSA (0.1 mg/ml, MedChemExpress, HY-P74175) into the otic vesicle of 3-dpf wild-type zebrafish embryos without MOVI modeling. 3 h later, we collected the whole body of each larva (*n*=80) for RNA isolation and performed qRT-PCR to detect the relative transcriptional expression levels of canonical IL-1 downstream target genes in zebrafish such as *nfkbia*, *ccl2*, *cxcl8*, *ptgs2* and *il1b*.

### Transcriptomics sample preparation

For RNA-seq analysis, the 3-dpf wild-type zebrafish larvae receiving DMSO or 10 μM AR-42 treatments were categorized into four groups: (1) DMSO-treated larvae without TFA modeling (referred to as DMSO), (2) AR-42-treated larvae without TFA modeling (referred to as AR-42), (3) DMSO-treated larvae with TFA modeling (DMSO_TFA) and (4) AR-42-treated larvae with TFA modeling (AR-42_TFA). There were four biological replicates per group. After TFA, larvae were immediately treated with DMSO or 10 μM AR-42 for 1 h before sampling. Tail tissues posterior to the cloaca in each larva were collected (*n*=150) and immediately frozen in liquid nitrogen before RNA isolation.

### RNA extraction, library preparation, sequencing and RNA-seq data analysis

Total RNA was extracted from zebrafish tail tissues using TRIzol Reagent (Invitrogen, 15596026). Qualified RNAs were finally quantified by Qubit3.0 with Qubit RNA Broad Range Assay kit (Life Technologies, Q10210). 2 μg total RNA was used for stranded RNA sequencing library preparation using KC-Digital Stranded mRNA Library Prep Kit for Illumina (DR08502, Wuhan Seqhealth, China) following the manufacturer's instructions. The library products corresponding to 200-500 bps were enriched, quantified and finally sequenced on a DNBSEQ-T7 sequencer (MGI Tech, China) with a PE150 model.

Qualified reads were mapped to the reference genome using STAR software (version 2.5.3a) and counted by featureCounts (Subread-1.5.1; Bioconductor). Genes differentially expressed between groups were identified using the edgeR package (version 3.12.1). A *P*-value cutoff of 0.05, fold-change cutoff of 2, and false discovery rate q-value cutoff of 0.05 were used to judge the statistical significance of gene expression differences. GO analysis and KEGG enrichment analyses for differentially expressed genes were both implemented by KOBAS software (version: 2.1.1) with a *P*-value cutoff of 0.05 to judge statistically significant enrichment. The RNA-seq analysis software and codes are listed in Table S2. Table S3 includes all the upregulated and downregulated genes and fragments per kilobase of transcript per million mapped reads (FKPM) values in all samples. The RNA-seq dataset has been deposited at the Gene Expression Omnibus (National Center for Biotechnology Information) with the accession number GSE240080.

### qRT-PCR

Total RNA was extracted using TRIzol and reverse transcribed using RT Master Mix for qPCR II (gDNA digester plus) (MedChemExpress). Real-time PCR amplification was performed using ChamQ Universal SYBR qPCR Master Mix (Vazyme) with a Bio-Rad CFX96 system according to the manufacturer's protocol. Relative expression was quantified by the 2-ΔΔct method. For the qRT-PCR validation of RNA-seq results, zebrafish larval tissue sample collection was performed according to the RNA-seq analysis (*n*=80). For detecting the IL-1 target gene expression in human IL-1β-injected zebrafish larvae, the whole bodies of larvae were collected for RNA isolation (*n*=80). The primer sequences for qRT-PCR are listed in Table S4.

### Mouse primary neutrophil isolation

As previously described (Ren et al., 2019), bone marrow was used to separate mouse neutrophils. Briefly, mouse bone marrow cells were harvested in Hanks-1 buffer [Hanks' balanced salt solution (HBSS) containing 0.5% bovine serum albumin and 10 mM HEPES], red blood cells were lysed in red blood cell lysis buffer (155mM NH_4_Cl, 10 mM KHCO_3_ and 0.1 mM EDTA at pH 7.4), and then discontinuous Percoll density gradient centrifugation was performed. Neutrophils were collected from the band that was distributed between 81% and 60% of Percoll. Cells were cultured in RPMI 1640 medium (Solarbio) containing 10% heat-inactivated fetal bovine serum and 25 ng/ml recombinant GM-CSF (PeproTech) with DMSO or AR-42 (10 μM) for 4 h before *in vitro* chemotaxis and spreading analyses.

### *In vitro* chemotaxis assay in a Dunn chamber

We monitored the chemoattractant gradient by free fluorescein isothiocyanate (FITC) dye, which has a similar molecular weight to N-formyl-Met-Leu-Phe (f-MLF, Sigma-Aldrich). AR-42-pretreated neutrophils were labeled with 0.05 μM calcium AM (Uelandy) and DMSO-pretreated neutrophils were not labeled. These two types of cells were then mixed at the ratio of 1:1 before being seeded onto a coverslip. Then, we imaged the neutrophil chemotaxis following the f-MLF gradient by using a Dunn chamber (DCC100, Hawksley) under a Leica DMI8 microscope. All time-lapse image series were acquired at 30 s intervals for 30 min and analyzed using the ImageJ plugin ‘Chemotaxis And Migration Tool’ (Schindelin et al., 2012).

We measured four chemotactic parameters: deflexion angle, Euclidean distance, accumulated distance and velocity. The deflexion angle represents the angle between the chemoattractant gradient direction and the direction of cell migration. The Euclidean distance, also known as displacement, is the straight-line distance of cell migration, whereas the accumulated distance is the route distance of neutrophil migration. f-MLF (5 μM) was used as a chemoattractant in this study.

### Spreading assay

Neutrophils were suspended in the assay buffer. Cells were seeded on polylysine-precoated slides to spread for 15 min, then fixed with 4% paraformaldehyde for 15 min. The slides were washed three times with PBS before imaging under a Leica DM6B microscope. The area of adherent neutrophils was quantified by using ImageJ (Schindelin et al., 2012).

### Neutrophil infiltration into inflamed peritonea *in vivo*

1 ml of autoclaved 3% thioglycollate medium (Sigma-Aldrich, T9032) was injected into the peritoneal cavity of mice to establish a mouse peritonitis model. Two intraperitoneal injections of AR-42 (25 mg/kg) or DMSO were performed 24 h before and 4 h post thioglycollate injection, respectively. 6 h after thioglycollate injection, mice were euthanized, and peritoneal lavage fluid was collected for analysis by flow cytometry. Neutrophils were identified by staining with a FITC anti-mouse CD45 antibody (1:500; Biolegend, 103108, lot B388747), an APC anti-mouse CD11b antibody (1:500; Biolegend, 101212, lot B384396) and a PE anti-mouse Ly-6G antibody (1:500; Biolegend, 127608, lot B307574).

### Statistical analysis

Statistical analysis was performed with GraphPad Prism 9 software. Data were analyzed using one-way ANOVA, two-way ANOVA or two-tailed unpaired Student's *t*-test. Data are displayed as mean±s.d. For the one-way ANOVA, post hoc tests such as Dunnett's test or Tukey's test were used, as specified in the figure legends. For the two-way ANOVA, the post-hoc Šídák's test was used, as specified in the figure legends. Differences were considered significant if *P*<0.05.

## Supplementary Material

10.1242/dmm.050056_sup1 Supplementary information

Table. S3. RNA-seq data. The Excel table file includes all the up-regulated and down-regulated genes and fpkm values in all samples.
