# Supplementary material for: Identification of histone deacetylase inhibitors as neutrophil recruitment modulators in zebrafish using a chemical library screen
Source: Dis Model Mech. 2023 Oct 13;16(10):dmm050056. doi: 10.1242/dmm.050056 (PMC10621070; doi:10.1242/dmm.050056)
Supplement: Supplementary information [file dmm-16-050056-s1.pdf]

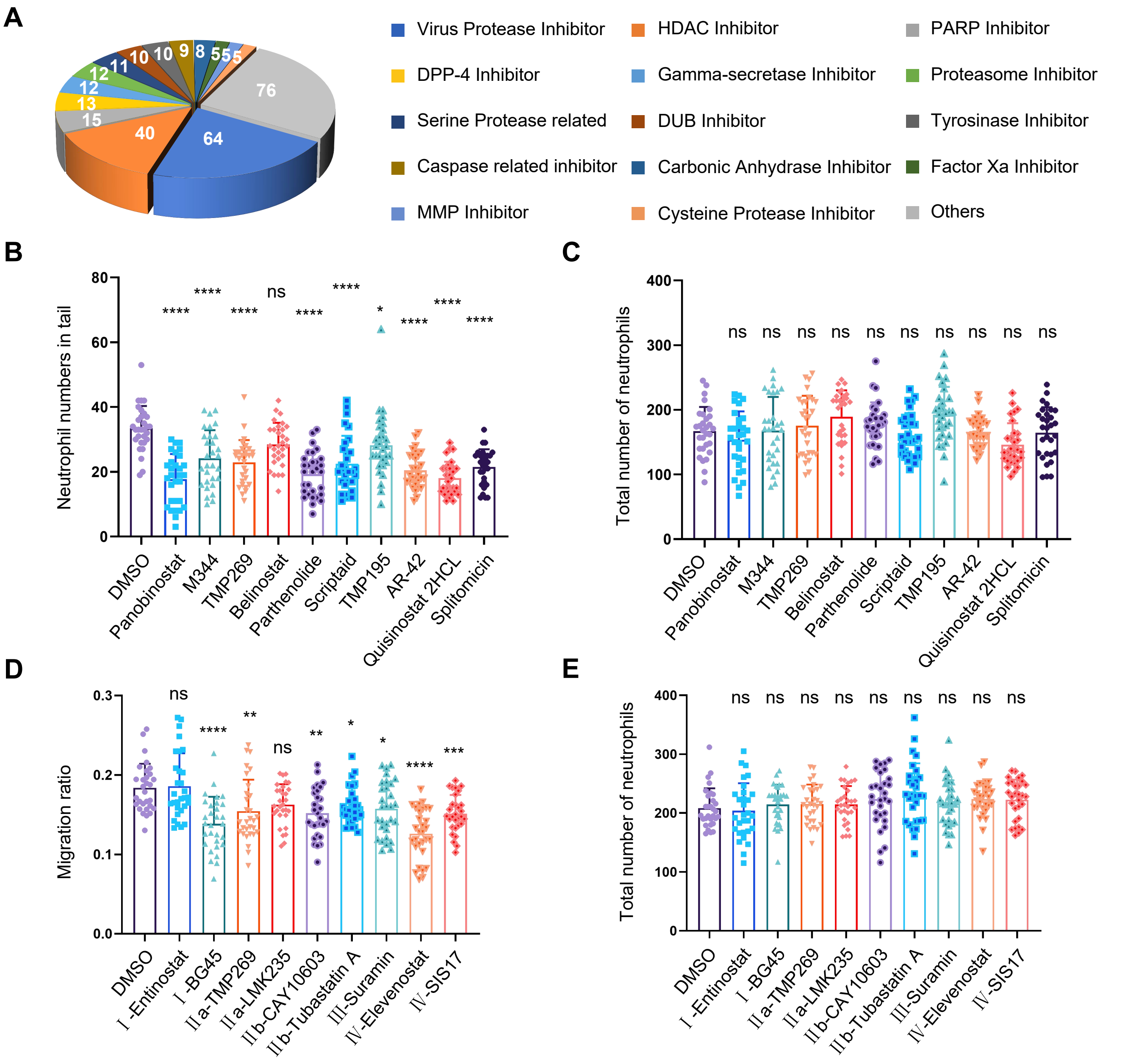

Fig. S1. HDAC Inhibitors from all four subfamilies modulate tissue injury-induced neutrophil recruitment.
(**A**) Classification of the 295 inhibitors targeting proteases and PTM enzymes.
(**B-C**) Quantitative analysis of GFP+ neutrophils in the tail region (**B**) and whole body (**C**) of 3-dpf *Tg(mpx:GFP)^i114^* embryos treated with indicated HDAC inhibitors in a TFA model (N = 30).
(**D-E**) Quantitative analysis of GFP+ neutrophils recruitment (migration ratio) (**D**) and total neutrophil numbers in the whole body (**E**) of 3-dpf *Tg(mpx:GFP)* embryos treated with indicated HDAC subfamily inhibitors in a TFA model (N = 30).
Each data point represents an individual embryo, One-Way ANOVA (Dunnett's test) for (**B-E**). Each HDAC inhibitor group was individually compared with the DMSO group. Error bars represent mean ± SD. *p < 0.05, **p < 0.01, ***p < 0.001, ****p < 0.0001. ns, not statistically significant. The experiments in (**B-E**) were repeated four times.

Disease Models & Mechanisms • Supplementary information

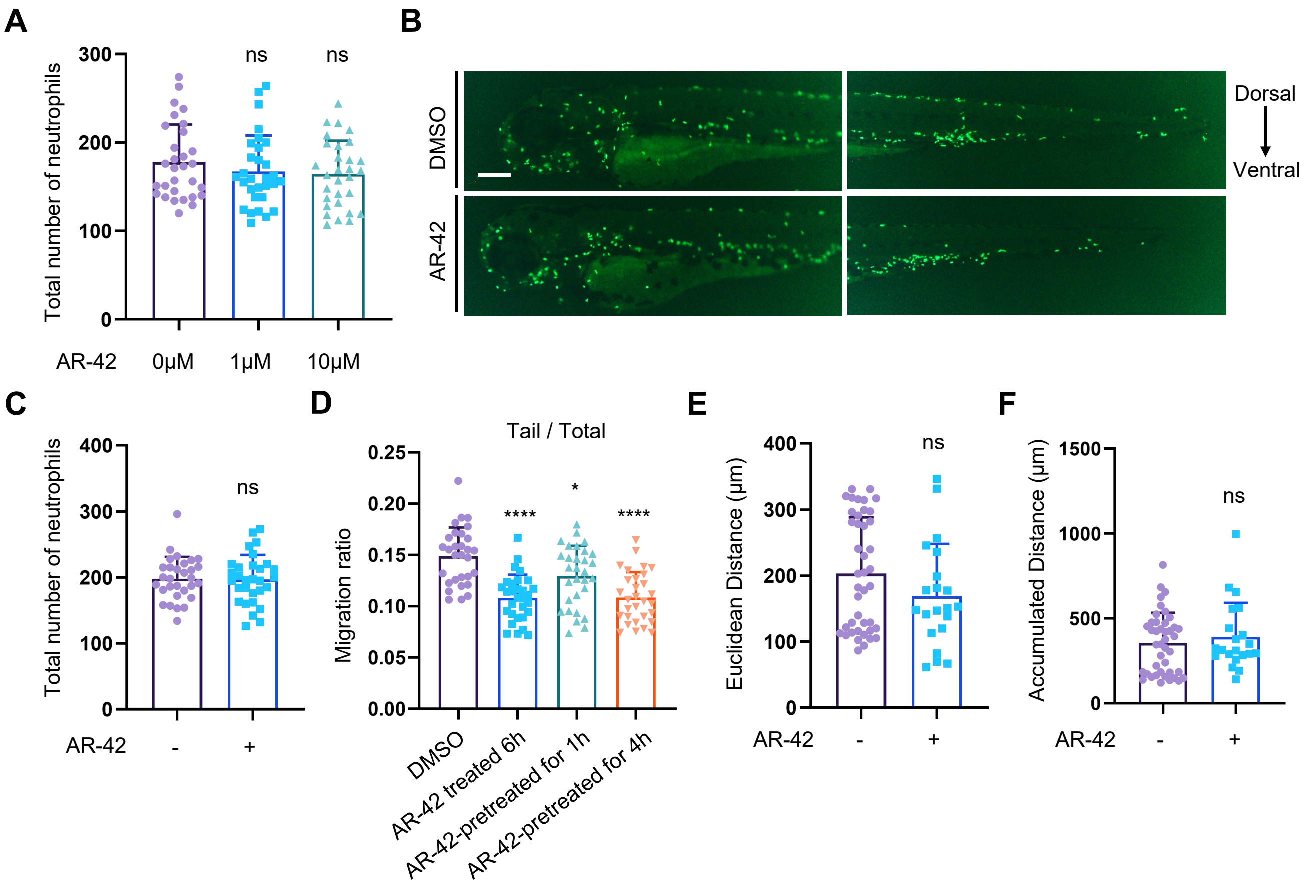

Fig. S2. AR-42 does not affect the total number of zebrafish neutrophils.

(**A**) Quantification of the number of whole-body neutrophils in AR-42 or DMSO-treated *Tg(mpx:GFP)*^{i114} embryos with TFA modeling, affilated to Fig. 2A (N = 30). Each data point represents an individual embryo, One-Way ANOVA (Dunnett's test). AR-42 treated group was individually compared with the DMSO group.

(**B-C**) Representative fluorescent images (**B**) and quantitative analysis (**C**) of total neutrophils in AR-42 or DMSO-treated zebrafish embryos without injury (N = 30). Scale bar = 100 μm. Each data point represents an individual embryo, student's t-test.

(**D**) Quantitative analysis of neutrophil recruitment to the wound area of 3-dpf *Tg(mpx:GFP)*^{i114} embryos treated with DMSO or AR-42 in the TFA model (N = 30). Three treatment conditions for AR-42: 6-h incubation after tail fin cut, 1-h and 4-h pretreatment before tail fin cut. Each data point represents an individual embryo, One-Way ANOVA (Dunnett's test). AR-42 treated group was individually compared with the DMSO group.

(**E-F**) Quantification of the euclidean distance (**E**) and accumulated distance (**F**) of neutrophils migrating toward the wound in Fig. 2C. Each data point represents a single cell, student's t-test. Error bars represent mean ± SD. $^*p < 0.05$, $^{****}p < 0.0001$. ns, not statistically significant. The experiments in (**A-D**) and (**E-F**) were repeated four and three times respectively.

Disease Models & Mechanisms • Supplementary information

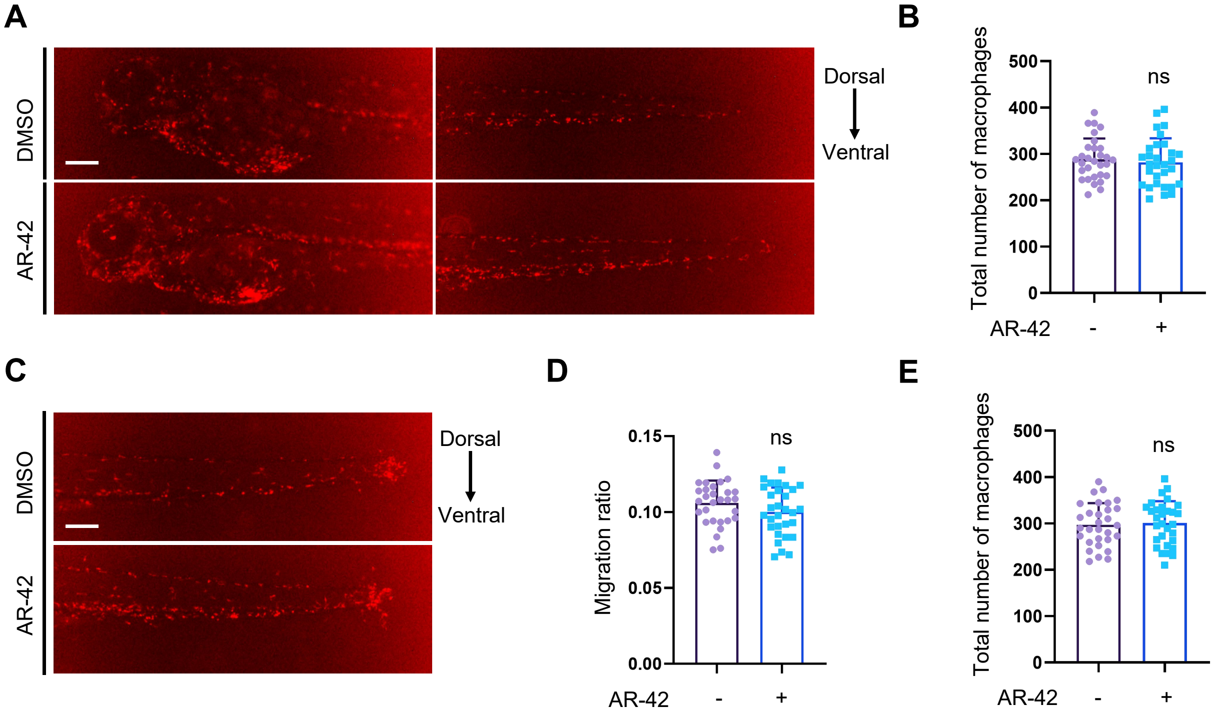

Fig. S3. AR-42 does not affect tissue injury-induced inflammatory macrophage migration in zebrafish.

(**A-B**) Representative fluorescent images (**A**) and quantitative analysis (**B**) of mCherry+ macrophages in DMSO or 10μM AR-42-treated 3-dpf *Tg(mpeg1:Gal4)gl24;Tg(UAS:Nfsb-mCherry)i149* zebrafish embryos without injury (N = 30). Scale bar = 100 μm.

(**C-E**) Representative fluorescent images (**C**) and quantitative analysis (**D-E**) of mCherry+ macrophages recruitment in DMSO or 10μM AR-42-treated 3-dpf *Tg(mpeg1:Gal4)gl24;Tg(UAS:Nfsb-mCherry)i149* zebrafish embryos with the TFA modeling. (N = 30). Scale bar = 100 μm. Each data point represents an individual embryo, student's t-test for (**B, D, E**). ns, not statistically significant. Error bars represent mean ± SD. The experiments in (**A-E**) were repeated four times.

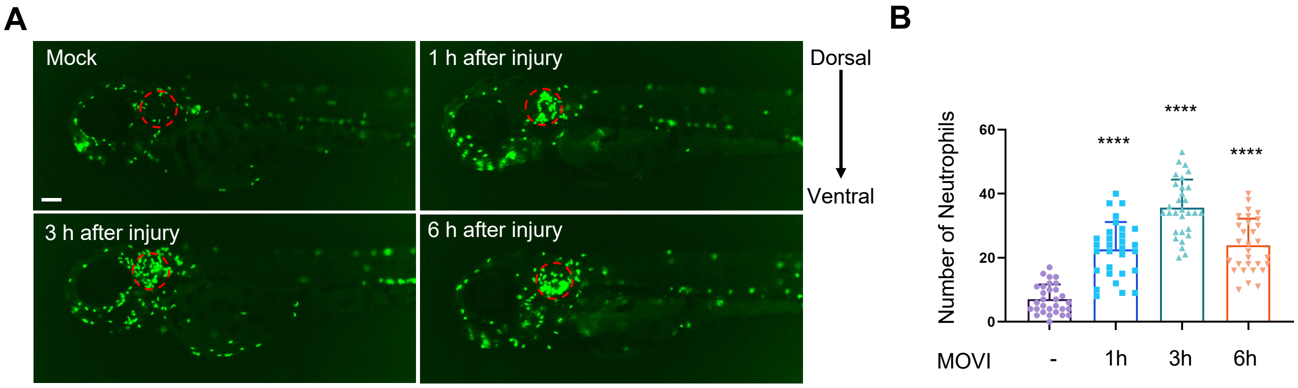

Fig. S4. Neutrophil influx reaches a peak at around 3 h after MOVI modeling.

(**A**) Representative fluorescent images of 3-dpf *Tg(mpx:GFP)*^{i114} embryos at 1, 3 and 6 h after MOVI modeling. The red dashed circle denotes the otic vesicle contour. Scale bar = 100 μm.

(**B**) Quantification of neutrophils recruited to the zebrafish otic vesicle in (**A**) (N = 30). Each data point represents an individual embryo, One-Way ANOVA (Dunnett's test). The modeling group was individually compared with the mock group.****$p < 0.0001$. Error bars represent mean ± SD. The experiments in (**A**-**B**) were repeated four times.

Disease Models & Mechanisms • Supplementary information

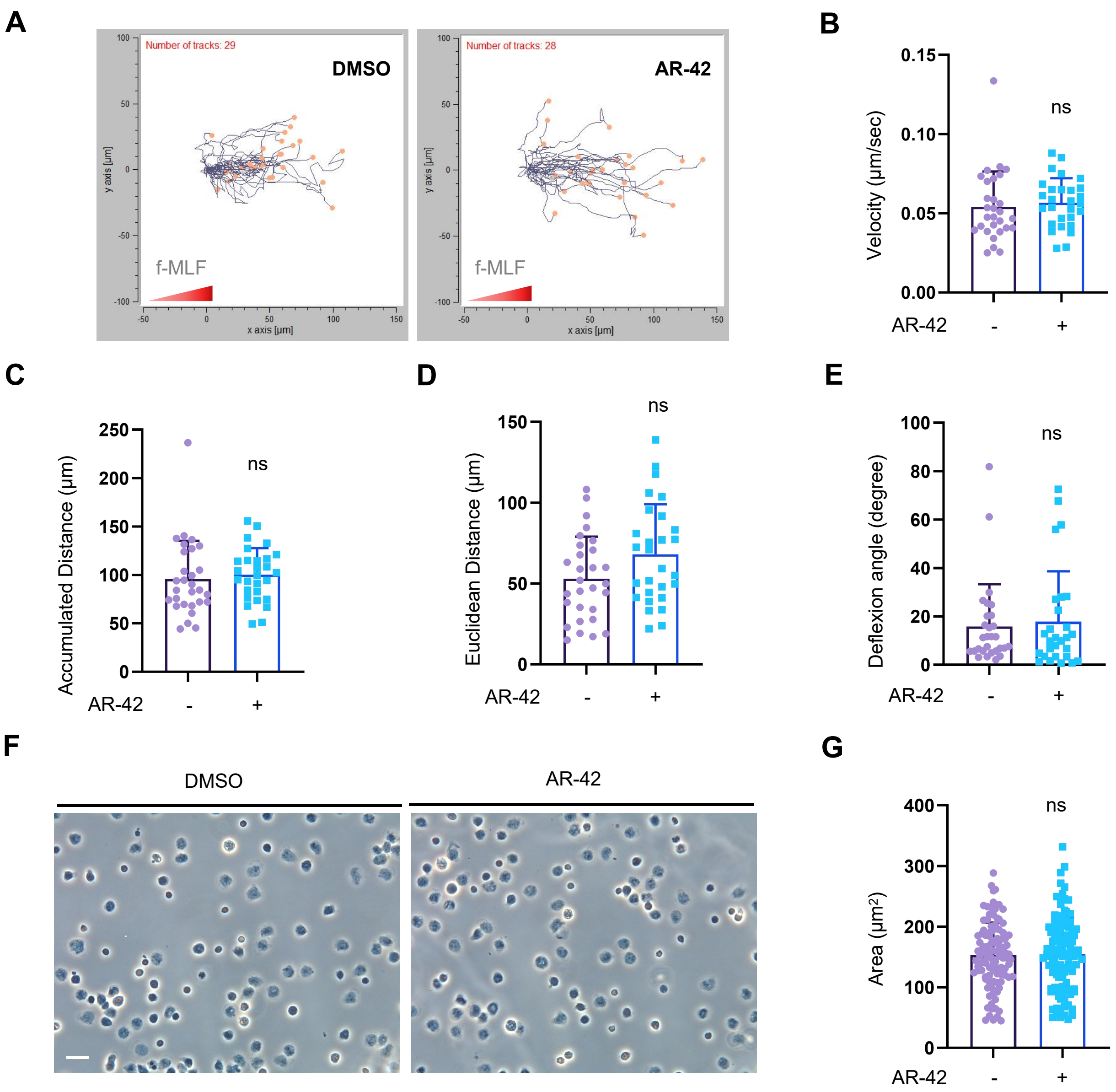

Fig. S5. AR-42 does not directly affect chemotaxis or spreading of mouse neutrophils *in vitro*.

(**A**) Representative migration trajectories of mouse neutrophil chemotaxis toward the f-MLF gradient in a Dunn Chamber analysis.(**B-E**) Quantification of the mean velocity, accumulated distance, euclidean distance, and deflexion angle of mouse neutrophil chemotaxis toward the f-MLF gradient in (**A**). Each data point represents an individual cell, student's t-test.

(**F-G**) Representative images (**F**) and quantification (**G**) of spreading of DMSO or AR42 pretreated neutrophils on poly-lysine pre-coated-slides (N = 100). Scale bar = 20 µm. Each data point represents an individual cell, student's t-test.

Error bars represent mean ± SD. ns, not statistically significant. The experiments in (**A-G**) were repeated three times.

Disease Models & Mechanisms • Supplementary information

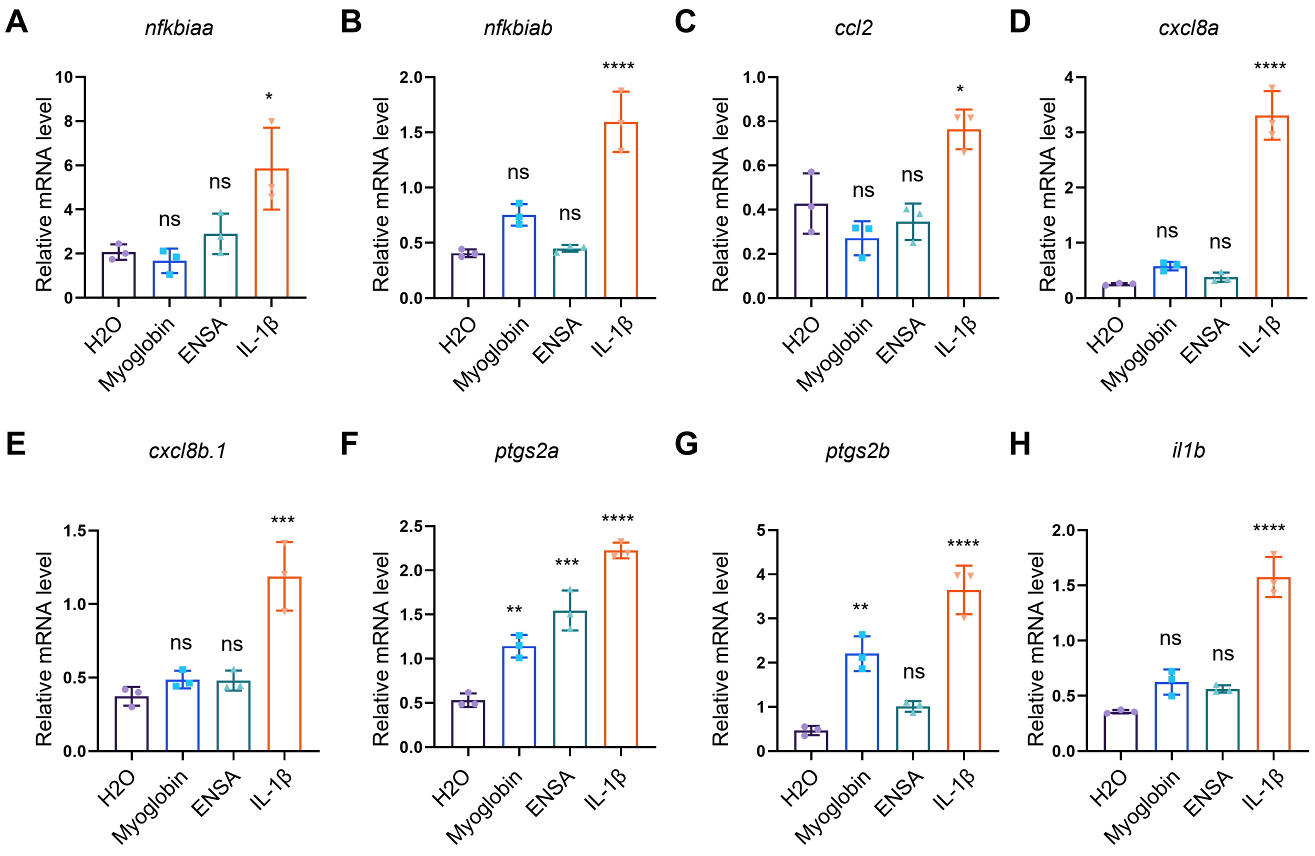

Fig. S6. Recombinant human protein IL-1β rather than Myoglobin or ENSA activates IL-1 signaling in zebrafish.

(**A-H**) qRT-PCR analysis of the expression level of *nfkbiaa*, *nfkbiab*, *ccl2*, *cxcl8a*, *cxcl8b.1*, *ptgs2a*, *ptgs2b* and *il1b* in 3-dpf Wild-type zebrafish embryos injected with 1 nL H2O or human recombinant proteins including Myoglobin, ENSA, IL-1β (0.1mg/mL) into the otic vesicle. Each data point represents a biological replicate, One-Way ANOVA (Tukey's test). Each recombinant protein group was individually compared with the H2O group. Error bars represent mean ± SD. **$p < 0.01$, ***$p < 0.001$, ****$p < 0.0001$. ns, not statistically significant. The experiments in (**A-H**) were repeated three times.

Table. S1. Classification of 41 inhibitors of proteases and PTM enzymes that significantly inhibited neutrophil recruitment in zebrafish.
Data were analyzed using One-way ANOVA (Dunnett's test). ****$p \leq 0.0001$, ***$p \leq 0.001$, **$p \leq 0.01$, *$p \leq 0.05$.

No.	Classification	Name	Target	Statistical significance
1	HDAC Inhibitors	Panobinostat	HDACs	****
2		MS344	HDACs	****
3		TMP269	Class IIa HDACs	****
4		Belinostat	HDACs	*
5		Parthenolide	HDAC1	****
6		Scriptaid	HDACs	****
7		TMP195	Class IIa HDACs	**
8		AR42	HDACs	****
9		Quisinostat 2HCL	HDACs	****
10		Splitomicin	Class III HDACs	****
11	Virus Inhibitors	Atazanavir sulfate	HIV Protease	*
12		Diphyllin	HIV Protease	***
13		Tenofovir alafenamide fumarate	HIV Protease	*
14		Paritaprevir	HCV Protease	**
15		Asunaprevir	HCV Protease	*
16		Mizoribine	HCV Protease	**
17	Caspase-related Inhibitors	PAC1	Caspase-3	**
18		Z-IETD-FMK	Caspase-8	**
19	PARP Inhibitors	Iniparib	PARP	*
20		Z-VAD-FMK	PARP	**
21	Proteasome Inhibitors	PI1840	Proteasome	***
22		Bortezomib-pinanediol	Proteasome	****
23	Gamma-secretase Inhibitors	RO4929097	Gamma-secretase	*
24		Avagacestat	Gamma-secretase	***
25	Tyrosinase Inhibitors	4-Butylresorcinol	Tyrosinase	*
26		Hexylresorcinol	Tyrosinase	**
27	Cysteine Protease Inhibitors	MG101	Cysteine Protease	*
28		2-cyano-Pyrimidine	Cysteine Protease	*
29	Others	Racecadotril	Neprilysin	***
30		Tanshinone IIA	VEGF / VEGFR2	**
31		P22077	USP7 / USP47	***
32		Licochalcone A	UGTs	**
33		ML281	STK33	****
34		IMR1	Notch	**
35		Betrixaban	Factor Xa	*
36		Teneligliptin hydrobromide	β2AR	*
37		Trelagliptin	DPP-4	*
38		Benzenesulfonamide	Carbonic Anhydrase	*
39		Dexibuprofen	COX	*
40		Calycosin	N/A	**
41		Glucosamine	N/A	*

Disease Models & Mechanisms • Supplementary information

Table. S2. RNA-seq Analysis softwares and codes.

Analysis Step	Software	Version	Description	Parameter
Raw data clean	Trimmomatic	version 0.36	Filter out the adaptor and low quality reads	ILLUMINACLIP:{adaptor.fa}:2:30:10 -phred33 LEADING:3 TRAILING:3 SLIDINGWINDOW:4:15 HEADCROP:0 MINLEN:36 PE
Clean data mapping	STAR	version 2.5.3a	mapping the reads to the genome	--outSAMtype BAM SortedByCoordinate
Gene expression calculation	featureCounts	Version 1.5.1	calculate the reads count for each gene	-T 10 -d 30 -D 1000 -C -s 1 -t {exon} -g {geneid} --primary -O -a {gff_annotation_file}
Differential expression analysis	edgeR	Version 3.12.1	Differential expression analysis	pvalue<0.05, |log2foldchang|>1
GO and KEGG enrichment	Kobas	Version 2.1.1	GO and KEGG enrichment	annotate.py: -t fasta:nuc -i -s -o, identify.py: -d K -f -o -p 0.05

Disease Models & Mechanisms • Supplementary information

Table. S3. RNA-seq data.

The Excel table file includes all the up-regulated and down-regulated genes and fpkm values in all samples.

Available for download at
https://journals.biologists.com/dmm/article-lookup/doi/10.1242/dmm.050056#supplementary-data

Table. S4. The primer sequences for qRT-PCR.

Primer	Sequence(5'-3')
Zebrafish *tuba1a*	CCTGCTGGGAACTGTATTGT
	TCAATGAGTTCCTTGCCAAT
Zebrafish *il1b*	CATTTGCAGGCCGTCACA
	GGACATGCTGAAGCGCACTT
Zebrafish *cxcl8a*	GTCGCTGCATTGAAACAGAA
	CTTAACCCATGGAGCAGAGG
Zebrafish *cxcl8b.1*	CCACACACACTCCACACACA
	CCACTGAATTGTCCTTTCATCA
Zebrafish *cxcl18b*	TGCCAGTGTGAAGAGTCATC
	TCGCAGTTTGGTCTTTTAGGG
Zebrafish *ptgs2a*	TGGATCTTTCCTGGTGAAGG
	GAAGCTCAGGGGTAGTGCAG
Zebrafish *ptgs2b*	GGGAGTCGCAGGTAAGAAAG
	GTGAGTAAAATGTTGGGCGAAG
Zebrafish *nfkbiaa*	CCTTGCCATCATTCACGAGG
	CTTTGCGTCTACATCTGCCC
Zebrafish *nfkbiab*	GCGGGTTGGATTCGTTAAAAG
	CGTGGATAATGGCGAGATGTAG
Zebrafish *ccl2*	GTCTGGTGCTCTTCGCTTTC
	TGCAGAGAAGATGCGTCGTA

Disease Models & Mechanisms • Supplementary information
